# Aal-circRNA-407 regulates ovarian development of *Aedes albopictus*, a major arbovirus vector, via the miR-9a-5p/*Foxl* axis

**DOI:** 10.1371/journal.ppat.1011374

**Published:** 2023-05-05

**Authors:** Yonghui Gao, Lu Yang, Yulan Chen, Peiwen Liu, Ying Zhou, Xiaoguang Chen, Jinbao Gu

**Affiliations:** Guangdong Provincial Key Laboratory of Tropical Disease Research, Department of Pathogen Biology, School of Public Health, Southern Medical University, Guangzhou, Guangdong, China; Institut Pasteur, FRANCE

## Abstract

*Aedes albopictus* shows a rapid global expansion and dramatic vectorial capacity for various arboviruses, thus posing a severe threat to global health. Although many noncoding RNAs have been confirmed to play functional roles in various biological processes in *Ae*. *albopictus*, the roles of circRNA remain a mystery. In the present study, we first performed high-throughput circRNA sequencing in *Ae*. *albopictus*. Then, we identified a *cysteine desulfurase* (*CsdA*) superfamily gene-originated circRNA, named aal-circRNA-407, which was the third most abundant circRNA in adult females and displayed a fat body highly expressed manifestation and blood feeding-dependent onset. SiRNA-mediated knockdown of circRNA-407 resulted in a decrease in the number of developing follicles and a reduction in follicle size post blood meal. Furthermore, we demonstrated that circRNA-407 can act as a sponge of aal-miR-9a-5p to promote the expression of its target gene *Foxl* and eventually regulate ovarian development. Our study is the first to report a functional circRNA in mosquitoes, expanding our current understanding of important biological roles in mosquitoes and providing an alternative genetic strategy for mosquito control.

## Author summary

In the present study, we performed high throughput circRNA sequencing and comprehensive analysis of circRNA profiles in adult males and females of A*edes albopictus*. CircRNAs were then confirmed to exist in this species. Based on several established circRNA studying tools applicable in mosquito species, we further identified a functional circRNA which is involved in the process of female ovarian development through miR-9a-5p/*Foxl* pathway. Overall, uncovering the biological functions of circRNAs in mosquitoes will provide novel and potential strategies for vector control.

## 1. Introduction

Circular RNAs (circRNAs) are single-stranded, covalently closed, endogenous noncoding RNAs (ncRNAs), and their distinct closed-loop structures are formed through a noncanonical “backsplicing” event by covalent bonds linking the 5’ and 3’ ends [1]. Therefore, this unique characteristic gives circRNAs more stability than linear RNAs and leads to a higher resistance to 3–5’ exonuclease RNase R (RNase R)-mediated degradation [2]. The circRNA was first identified as a plant viroid in 1976 [3] and was first observed in human HeLa cells by electron microscopy in 1979 [4]. In recent years, with the rapid development of high-throughput RNA sequencing (RNA-seq) and bioinformatics-based circRNA detection algorithms, numerous circRNAs have been identified and annotated in all domains of life, including viruses [5], prokaryotic archaea [6] and eukaryotes, such as yeast, fungi, plants, protists, worms, insects, fish and mammals [7].

For a long time, circRNAs have typically been considered aberrant RNA splicing byproducts with little functional potential [8]. However, with the rapid improvement of circRNA-specific sequencing and quantitative analysis methods, many circRNAs are detected as the predominant transcript compared to their linear counterparts [9], indicating that circRNAs have potential functions that reflect their higher stability, high abundance, and high conservation. Although the biological functions of circRNAs are not completely understood to date, several major mechanisms of circRNA functions have been elucidated. For example, the most prominent function of circRNAs is their action as competing endogenous RNAs (ceRNAs) to sequestermiRNAs and inhibit their activity [10]. CircRNAs can also act as RNA-binding protein (RBP) sponges to regulate downstream gene transcription [11]. AUG circRNAs can act as mRNA traps that regulate host gene expression by sequestering the translation initiation site [12]. Some circRNAs are predominantly distributed in the nucleus and act as DNA replication regulators by pairing with DNA to form DNA-RNA triple helices at the transcription initiation site [13]. Some circRNAs can also encode peptides or proteins using cap-independent, internal ribosome entry site (IRES) or N6-methyladenosine (m^6^A)-mediated translation initiation [14]. Several circRNAs can serve as protein scaffolds or are recruited by facilitating interactions between proteins or recruiting specific proteins to certain loci or subcellular compartments [15]. CircRNAs can be divided into four main subtypes, exonic circRNAs (EcircRNAs), circular intronic RNAs (ciRNAs), exonic-intronic circRNAs (EIciRNAs) and tRNA intronic circRNAs (TricRNAs), depending on four proposed biogenesis pathways: lariat-driven circularization, RNA-binding protein-mediated circularization, direct back-splicing and tRNA splicing enzyme-mediated circularization [16].

Compared to the comprehensive exploration of circRNAs in mammals, research on circRNAs in insects is still in its infancy. The genome-wide identification of insect circRNAs was first performed in *Drosophila melanogaster* in 2014 [17]. Subsequently, a relatively comprehensive identification of circRNAs was performed in *Bombyx mori* [18], *Apis mellifera* [19], *Dendrolimus punctatus* [20], *Helicoverpa armigera* [21] and *Culex pipiens pallens* [22]. Nevertheless, only a few circRNAs have been functionally characterized. For example, an insulin-sensitive circRNA influences *Drosophila* lifespan [23], a *histone-lysine N-methyltransferase eggless* (*BmEgg*) gene-derived circRNA (circEgg), regulates histone H3K9me3 by sequestering bmo-miR-3391–5p and encoding circEgg-P122 protein in silkworms. To date, however, the characteristics and biological roles of circRNAs in mosquitoes remain a mystery.

The Asian tiger mosquito, *Aedes albopictus* (Skuse) (Insecta: Diptera: Culicidae), a mosquito originally from southeast Asia, shows a dramatic global expansion and was listed as one of the ’100 of the World’s Worst Invasive Alien Species’ [24]. *Ae*. *albopictus* has been shown to be a competent vector that can experimentally transmit at least 26 arboviruses that have spread throughout the world [25], which ranks as a secondary vector of dengue virus in the world and primary vector in mainland China [26].

In the present study, we first performed high-throughput circRNA sequencing (circRNA-seq) and comprehensive analysis of circRNA profiles in adult males and females of *Ae*. *albopictus* based on the detection of back-spliced junction (BSJ) reads. We identified a *cysteine desulfurase* (*CsdA*) superfamily gene (National Center for Biotechnology Information (NCBI) GenBank No: *LOC109412837*)-originated circRNA, named aal-circRNA-407, which was the third most abundant circRNA in adult females and displayed a fat body-enriched manifestation and blood feeding-dependent onset. SiRNA-mediated knockdown of circRNA-407 resulted in a decrease in the number of developing follicles and a reduction in follicle size during ovarian development. By establishing several circRNA study methods applicable in mosquito species, we further demonstrated that circRNA-407 can act as a sponge of aal-miR-9a-5p to promote the expression of its target gene *Foxl* (*forkhead box protein L*) and eventually regulate ovarian development. Overall, our study first reported a functional circRNA in mosquitoes that is involved in the process of female oogenesis via the miR-9a-5p/*Foxl* pathway, expanding our current understanding of the important roles of circRNAs in biological processes in mosquitoes and providing a reference for further exploration of circRNAs in mosquitoes.

## 2. Results

### 2.1 The workflow of RNA-seq and data processing

**Fig 1:**
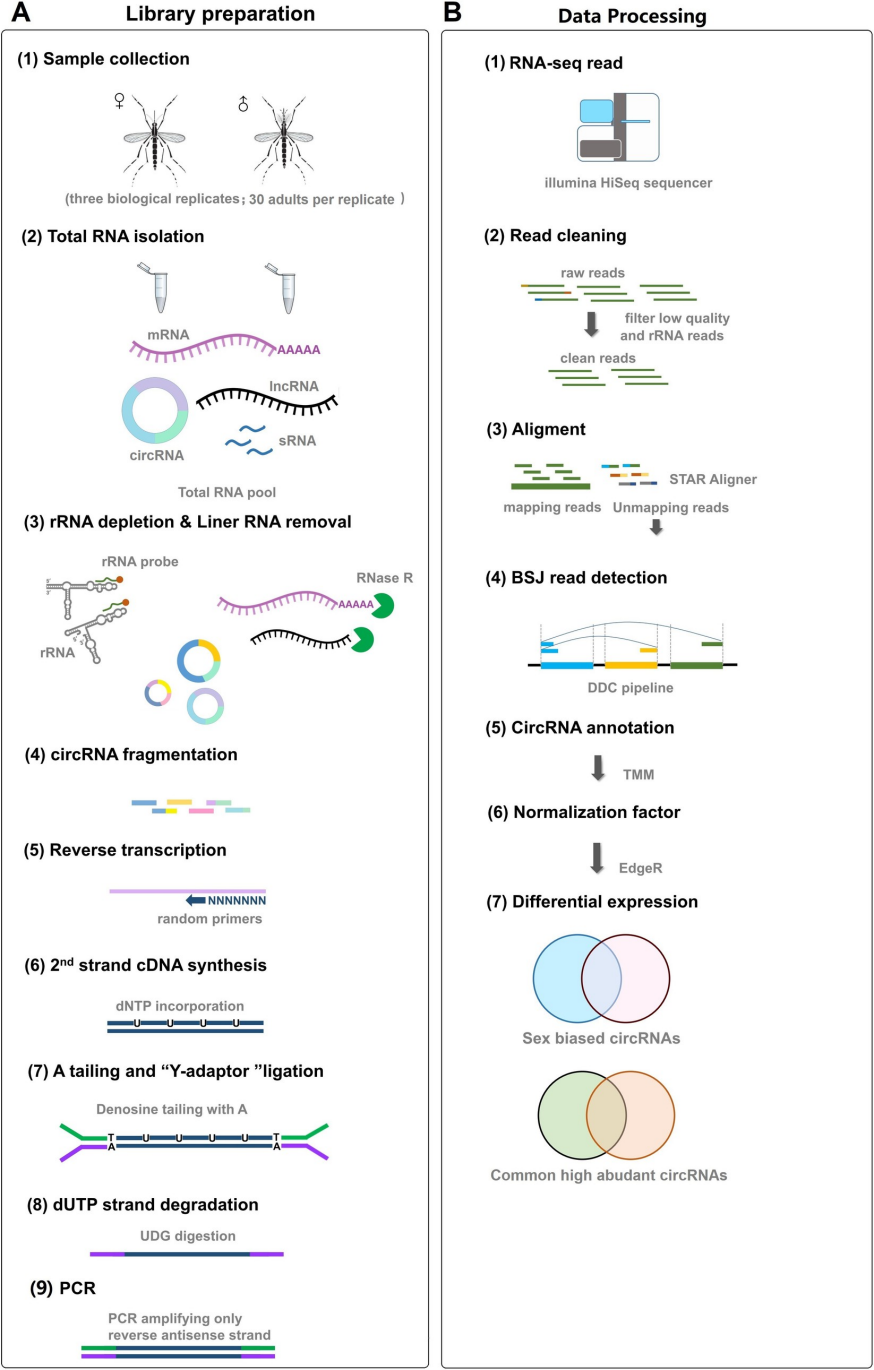
Overview of sample curation, circRNA-seq and Computational Data Analysis pipeline. **(A)** Preparation of circular RNA (circRNA) libraries for circRNA sequencing. Total RNA was extracted from 2 days post emergence virgin adult females and males, respectively. Then, circRNAs were enriched via ribosomal RNA (rRNA) depletion and linear RNA removal. Next, circRNAs were fragmented and reverse transcribed. Subsequently, second strand cDNA was synthesized and ligated Illumina sequencing adaptors. Finally, the second strand of cDNA was digested and the libraries were subjected to paired-end sequencing on an Illumina HiSeq sequencer. **(B)** Processing of circRNA sequencing data. Raw paired end Illumina data were cleaned by Trimmomatic. Next, all clean reads were mapped to the *Ae*. *albopictus* Foshan strain reference genome using STAR. Then DCC was applied to detect and quantify putative circRNAs in all sample groups. Differentially expressed and common high abundant circRNAs between adult females and males were estimated respectively using the edgeR package followed by CPM (counts of exon model per million mapped reads) normalization.

The workflow of RNA-seq and data processing of the two libraries is shown in Fig 1, and the details of the methods used for rRNA- and RNase R+ library preparation and data processing are described in the Materials and Methods.

### 2.2 Characteristics of circRNA profiles in adult *Ae*. *albopictus*

**Fig 2:**
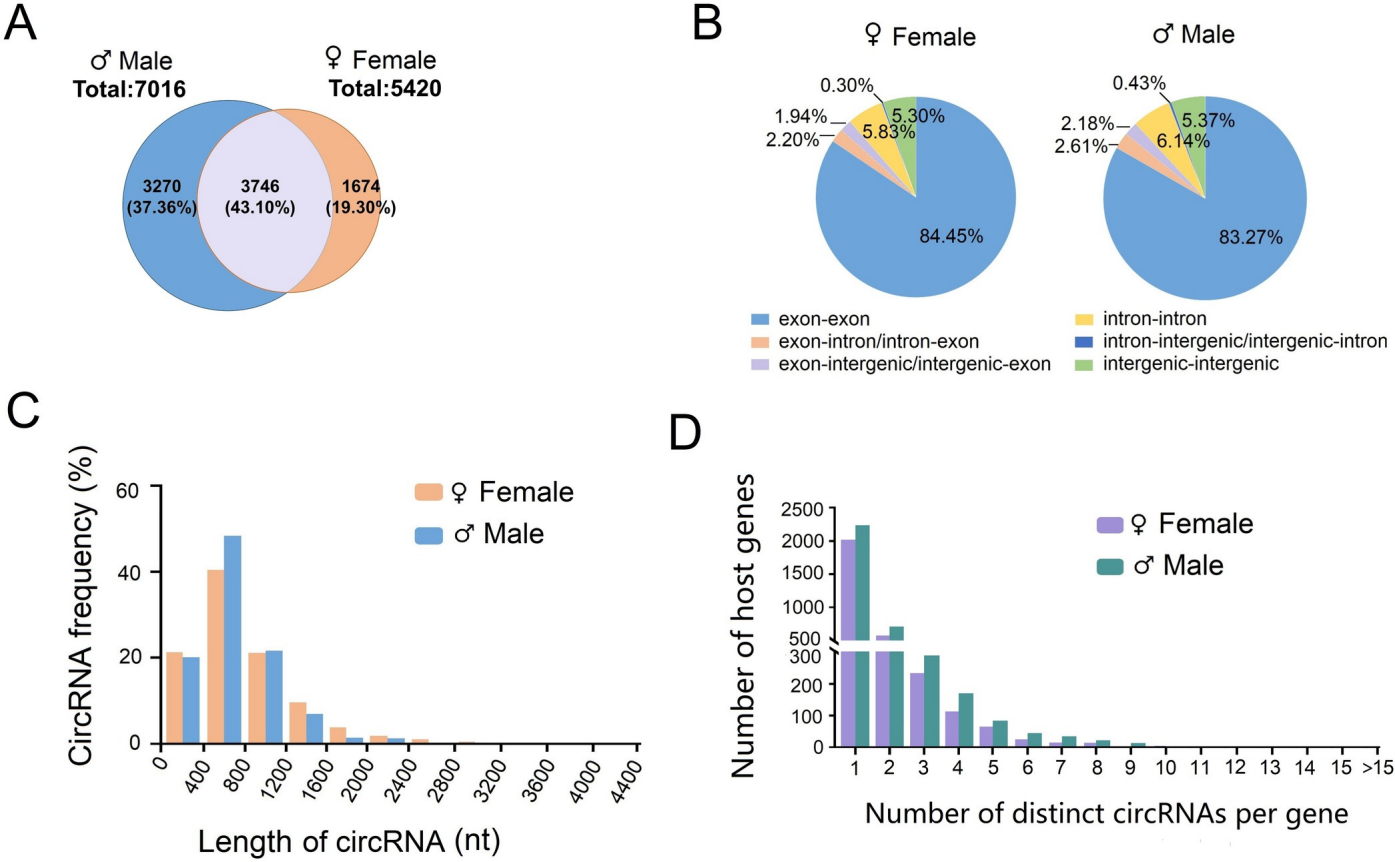
General characteristics of circRNAs in *Ae*. *albopictus* adults. **(A)** Venn diagram showing the unique and shared circRNAs between adult females (n = 3) and males (n = 3). **(B)** Pie chart indicating the distribution of genomic regions of the *Ae*. *albopictus* circRNAs recognized by circRNA sequencing. CircRNAs were divided into the following six categories according to genomic origin of the BSJ reads: exon-exon, exon-intron/intron-exon, exon-intergenic/intergenic-exon, intron-intron, intron-intergenic/intergenic-intron and intergenic-intergenic. **(C)** Length distribution of circRNAs. **(D)** Number of distinct circRNA candidates per gene.

To examine the circRNA expression profiles of *Ae*. *albopictus*, we profiled circRNAs in female and male mosquitoes. rRNA depleted total RNA-Seq libraries were prepared and sequenced with paired-end reads. After removing adapter-containing reads, poly-N-containing reads and low-quality reads, a total of 127,640,747 and 56,842,626 clean reads were obtained from the female and male mosquito libraries (three replicate libraries per sex). Cleaned reads were then aligned to the *Ae*. *albopictus* Foshan strain reference genome *Aalbo_primary*.*1* with STAR. As a result, a total of 7,016 and 5,420 circRNA candidates were identified in adult females and males, respectively, with at least one head-to-tail junction read in one group. Among them, 3,746 circRNAs were found to be common in females and males, while 3,270 circRNAs were unique in females, and 1,674 circRNAs were specifically present in males (Fig 2A). All predicted circRNA junction reads in each group are listed in S2 Table. Based on the location of the BSJ site on the genome, these circRNAs were divided into six types: exon-exon, exon-intron/intron-exon, exon-intergenic/intergenic-exon, intron-intron, intron-intergenic/intergenic-intron and intergenic-intergenic (Fig 2B). The lengths of most exonic circRNAs (approximately 76.13%) were no more than 1000 nt, and the median length was 400–800 nt (Fig 2C). Furthermore, 2,010 (65.69%) and 2,214 (61.60%) circRNA-producing genes derived more than one circRNA in females and males, respectively (Fig 2D).

### 2.3 circRNA expression analysis in adult *Ae*. *albopictus*

**Fig 3:**
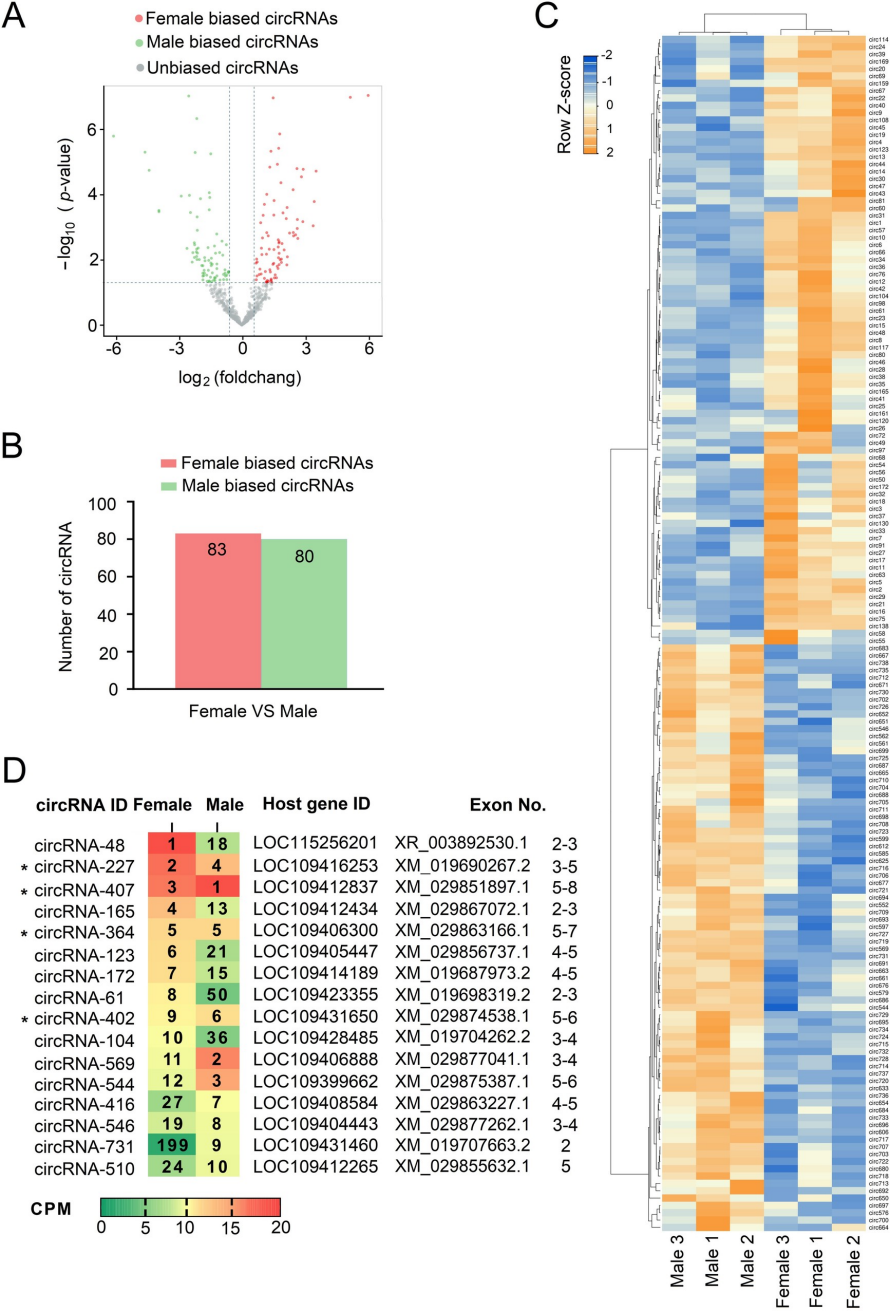
Sex-biased expressed and common top high abundant circRNAs in adult females and males. **(A)** and **(B)** Volcano plots and bar graph showing significant differentially expressed circRNAs between female and male *Ae*. *albopictus*. The red circles/bar denoted female biased circRNAs, green circles/bar denoted male biased circRNAs and grey circles denoted non-biased circRNAs. **(C)** Hierarchical cluster analysis of the CPM-flittered circRNAs in six sequencing sample groups. The dendrogram showed the relationships among the expression levels of samples. CircRNAs with | (log_2_FC) | > 1 and p-value <0.05 were considered to be sex-biased circRNAs. **(D)** The top 10 abundant circRNAs in female and male groups on the basis of CPM values. Red color indicates a higher expression level of circRNAs and green color indicates lower expression level. The numbers in each column represented the expression level of circRNA in descending order. Asterisk indicated the four circRNA candidates which ranked among the top 10 most abundant circRNAs in both female and male groups.

Since expression analysis of circRNAs is not affected by length of circRNA but the library sizes of the samples, we normalized circRNA expression by CPM method to avoid the biases derived from different library insert size and sequencing depth [27]. Those circRNAs for which the CPM values were less than 0.5 in half of the samples were filtered out. Then, a total of 738 filtered circRNAs were used for further differential expression analysis (S2 Table). With the threshold of |log_2_(fold change)| >1, *p* value <0.05, 163 circRNAs were identified as having a sex-biased expression pattern between the females and males, of which 83 showed a higher expression pattern in females, and 80 were found to be significantly higher in males. CircRNA expression variation between females and males is represented as volcano plots (Fig 3A), bar graphs (Fig 3B), and heatmaps (Fig 3C). Meanwhile, presuming that circRNAs that are highly expressed are thought to be more likely to be involved in important or crucial biological functions [28], we also listed the top ten abundant circRNAs in females and males (Fig 3D). We noticed that four circRNAs, aal-circRNA-227, aal-circRNA-407, aal-circRNA-364 and aal-circRNA-402, were commonly ranked among the top 10 most abundant circRNAs in both the female and male groups. Since it has also been reported that most mammalian circRNAs are largely non-functional products of splicing errors [29], in the present study, we mainly focused on circRNAs that were abundant in both female and male mosquitoes. Therefore, these four circRNAs were selected for subsequent analyses.

### 2.4 Experimental Validation of *Ae*. *albopictus* circRNAs

**Fig 4:**
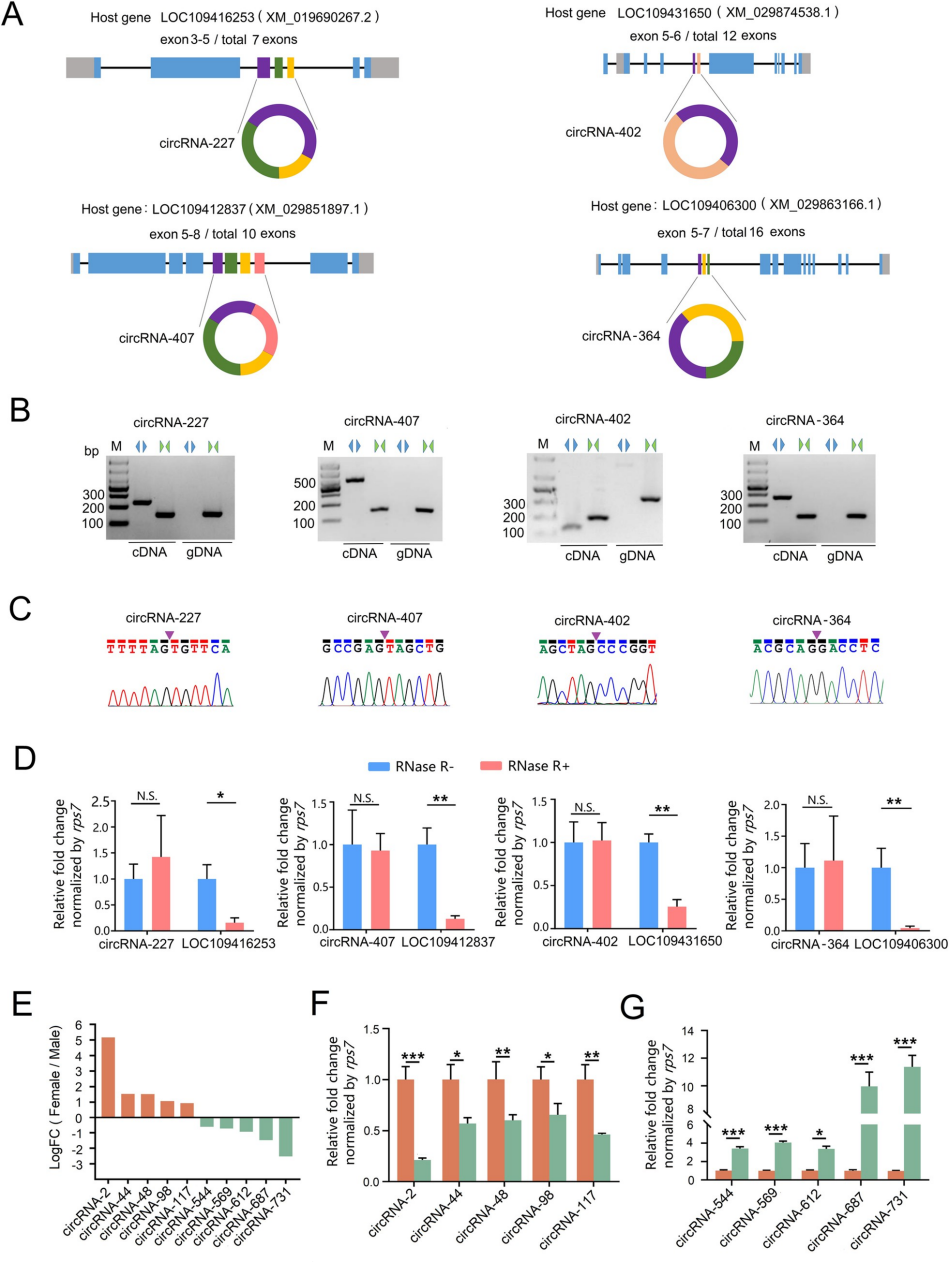
Experimental validation of circRNA candidates. **(A)** Schematics of the biogenesis of circRNA candidates and gene structure of their host genes. Black line represents intron, whereas rectangle indicates exon. Gray rectangle represents the UTR region, differently colored rectangles suggests the circularized exons. Exons and introns are not shown to scale. **(B)** Divergent primers successfully amplified the corresponding bands in cDNA templates but failed in genomic DNA (gDNA). Convergent primers worked with both cDNA and genomic DNA. Blue back-to-back triangle pairs represents divergent primers and green face-to-face triangle pairs represents convergent primers. **(C)** Sanger sequencing of amplification using divergent primers in the cDNA templates confirmed the BSJ of circRNA candidates predicted by circRNA-seq. Red inverted triangles indicates the BSJ. **(D)** Resistance of circRNA candidates to RNase R digestion determined by qRT-PCR. Red bars represents RNaes R treated groups whereas blue bars represents negative controls. Relative expression level of circRNA candidates and their host gene mRNAs in negative controls was set as 1. **(E)** Log_2_ fold change (Females V.S. Males) of the selected sex-biased circRNAs from circRNA-seq expression analysis. **(F)** and **(G)** qRT-PCR validation for female-biased circRNAs (F) and male-biased circRNAs (G). Relative expression level of circRNAs in females was set as 1. All qRT-PCR were performed triplicates with three biological replicates (n = 15 adults per replicate), values were presented as means ± SEM. Student’s t-test was used to compare the means between two groups. *p < 0.05; **p < 0.01; ***p < 0.001; N.S., no significance.

To confirm the sequencing data of mosquito circRNA prediction and identification, we first designed divergent and convergent primers to amplify the four candidate circRNAs and their corresponding linear host mRNAs using cDNA and genomic DNA (gDNA) templates, respectively (Fig 4A). The amplified PCR products using divergent primers were further analyzed by Sanger sequencing to confirm the presence of the back-splicing junctions. As shown in Fig 4B, all divergent primers could only amplify a single band in the cDNA template, whereas the convergent primers could amplify corresponding bands in both the cDNA and gDNA templates. The Sanger sequencing results further verified the presence of head-to-tail junctions predicted by circRNA-seq. Furthermore, to determine the resistance of the circRNAs to Rnase R cleavage, total RNA extracted from female mosquitoes was treated with Rnase R prior to reverse transcription. The expression levels of circRNAs and their linear host mRNAs in Rnase R-treated samples were further analyzed by qRT-PCR. As a result, compared with the no RNase R treatment control, the expression of all tested circRNAs was not affected by RNase R digestion, while their corresponding linear mRNA controls were dramatically depleted, confirming the circularity of circRNAs (Fig 4D). Furthermore, to verify the accuracy of circRNA sequencing and differential expression analysis, we chose circRNAs that were either female or male biased (Fig 3B) for qRT-PCR analysis. First, the expected BSJ were confirmed by Sanger sequencing (S1A and S1B Fig). Then, these ten circRNAs were subjected to qRT-PCR analysis using the specific primers. As shown in Fig 4E–4G, the results from the RT-qPCR for ten sex biased circRNAs was consistent with the trends obtained from the circRNA sequencing analysis, demonstrating that the circRNA sequencing result and expression levels of candidate circRNAs are reliable in the present study.

### 2.5 Spatial and temporal expression patterns of circRNA candidates

**Fig 5:**
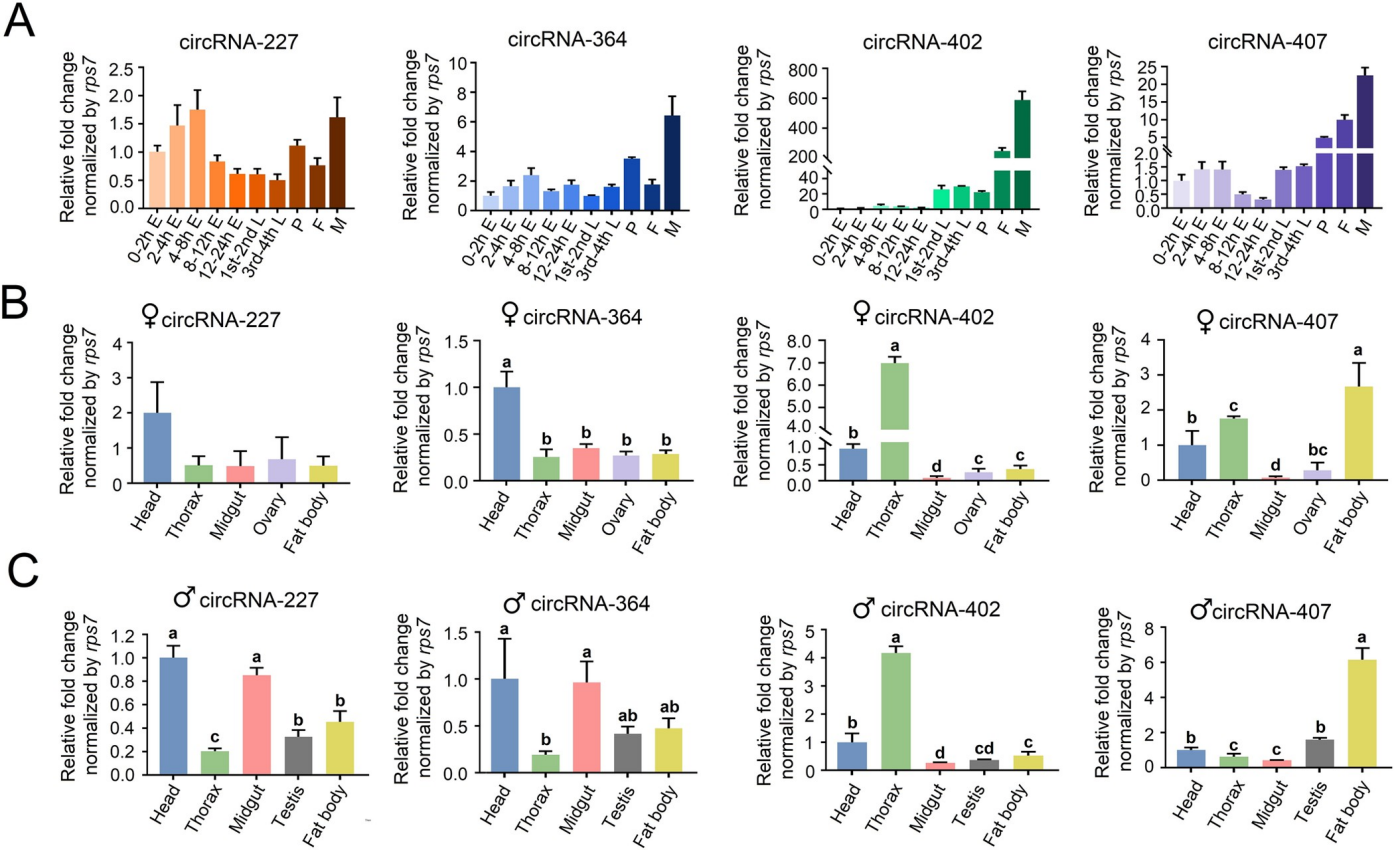
Spatial-temporal patterns of four high abundant circRNA candidates measured by real-time RT-PCR. **(A)** Temporal profiles of circRNA candidates at different developmental stages of *Aedes albopictus* determined by qRT-PCR. E = n hours post-oviposition embryo (n = 200 per replicate); 1st -2nd L = 1st -2nd instar larvae; 3rd-4th L = 3rd-4th instar larvae (n = 40 per relicate); P = pupae (n = 20 per replicate); F = female (n = 15 per replicate); M = male (n = 15 per replicate). Relative expression level of circRNA candidates in 0-2h post-oviposition embryo was set as 1. **(B)** and **(C)** Spatial expression patterns of circRNA candidates in different tissue of *Ae*. *albopictus* adult females and males (n = 15 adults per replicate) determined by qRT-PCR. ♀ = adult females; ♂ = adult males. Relative expression level of circRNA candidates in head was set as 1. The X-axis indicated the different sample groups and the Y-axis showed the relative expression levels. All qRT-PCR were performed triplicates with three biological replicates, values were presented as means ± SEM. One-way ANOVA was used to compare the means among different groups and different letters above the bars denote statistical significance at *p* < 0.05.

To analyze the temporal expression profiles of the four candidate circRNAs, total RNA extracted from various embryonic stages to adults was reverse-transcribed by random primers. qRT-PCR analysis showed that these 4 circRNAs displayed different expression patterns (Fig 5A). Briefly, circRNA-227 showed the highest relative expression level in the embryonic stages, reaching its maximum in embryos 4–8 hours post oviposition, whereas circRNA-227 increased again in adults. CircRNA-364, circRNA-402 and circRNA-407 showed distinctly higher levels in adult stages.

The spatial expression pattern of these four circRNAs in *Ae*. *albopictus* was also quantified by performing qRT-PCR on total RNA extracted from different tissues, including head, thorax, midgut, ovaries (in females) or testis (in males) and fat bodies dissected from adult mosquitoes 72 h PE. As shown in Fig 5B and 5C, all circRNA candidates showed relatively obvious tissue-enriched expression patterns. CircRNA-227 and circRNA-364 showed greater expression levels in the head, and circRNA-402 was predominantly expressed in the thorax, whereas circRNA-407 displayed significantly higher expression in the fat body.

### 2.6 Expression profiles of circRNA-407 in the fat body of female mosquitoes during vitellogenesis

**Fig 6:**
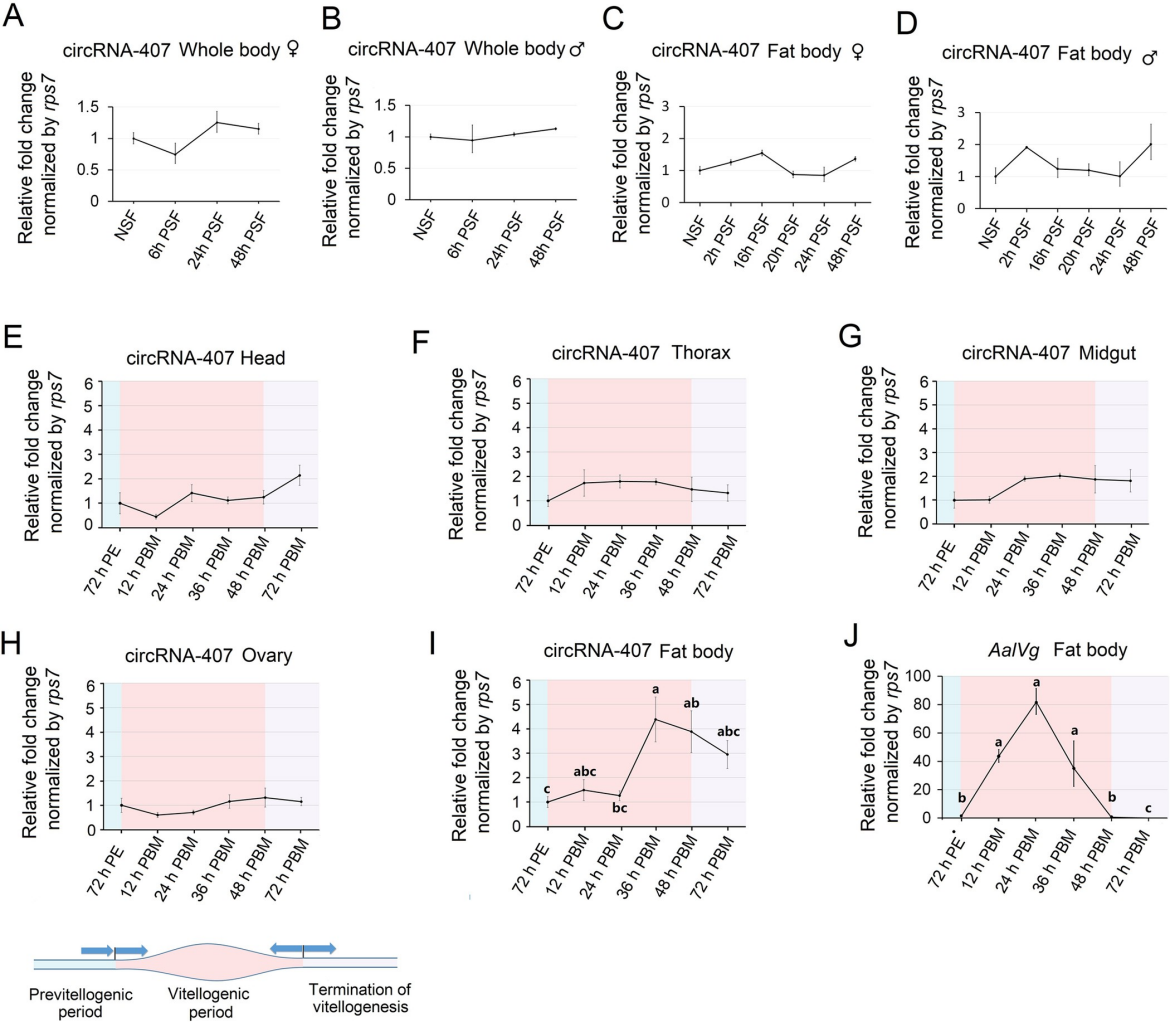
Expression profiles of circRNA-407 in mosquito adults upon sugar or blood meal stimulation. **(A)** to **(D)** Dynamic expression levels of circRNA-407 in whole body or fat body of adult females or males (n = 15 adults per replicate) after a sugar meal determined by qRT-PCR. NSF = non-sugar fed; PSF = hours post sugar fed. Relative expression level of circRNA-407 in NSF groups was set as 1. **(E)** to **(I)** Dynamic expression levels of circRNA-407 in different tissue of adult females (n = 15 per replicate): Head(E), Thorax(F), Midgut(G), Ovary(H) and Fat body (I) at different time points after a blood meal determined by qRT-PCR. PE = hours post-emergence; PBM = hours post blood meal. Relative expression level of circRNA-407 in 72 h PE groups was set as 1. Different background colors indicated three phases of female mosquito oogenesis cycle. **(J)** Expression levels of *AalVg* determined by qRT-PCR was used to indicate the physiological stage of vitellogenic female mosquitoes. All qRT-PCR were performed triplicates with three biological replicates and values were presented as means ± SEM. One-way ANOVA was used to compare the means among different groups and different letters above the bars denote statistical significance at *p* < 0.05.

Among the four circRNA candidates, we noticed that circRNA-407, which was among the top 3 abundant circRNAs in both the female and male groups in our circRNA-seq data (Fig 3D), exhibited a fat body-enriched expression pattern. Fat bodies have been studied to play a crucial and complex role in biological processes of mosquitoes, such as nutrient metabolism, lipid storage, and amino acid transportation [30]. To explore whether fat body-enriched circRNA-407 was involved in various physiological events in the mosquito fat body, we first examined the dynamic changes of circRNA-407 during glycometabolism. Briefly, newly emerged adult females and males were given a 10% glucose meal, and then fat bodies from each sugar-fed mosquito were dissected at different time points postsugar feeding (PSF) according to previous research. The expression levels of circRNA-407 in the fat body at different time points postsugar feeding were analyzed by qRT-PCR. As shown in Fig 6A–6D, circRNA-407 remained at a stable level during digestion of sugar, suggesting that circRNA-407 may not be involved in glucose metabolism in the fat body of adult mosquitoes. However, females also need a blood meal for ovarian development and reproduction. Ovarian development of female mosquitoes is a complex physiological process, and fat bodies play a critical role in blood meal metabolism and vitellogenesis [31–32]. Therefore, 72 h PE female mosquitoes were provided blood meal, and different tissues, including head, thorax, midgut, ovary and fat body, were dissected and collected at different time points post blood meal, and the dynamic expression of circRNA-407 during ovarian development was performed by qRT-PCR as described above. Meanwhile, the transcriptional level of *Ae*. *albopictus vitellogenin-A* (*VgA*, NCBI GenBank No. *LOC109413693*) in the fat body was used to represent the different physiological phases of vitellogenic female mosquitoes. As a result, no significant expression changes in circRNA-407 were observed in the head, thorax, midgut or ovary after a blood meal. In the fat body, however, the transcriptional level of circRNA-407 suddenly increased to nearly 4.5 times the transcriptional level at 36 h PBM and then decreased gradually until 72 h PBM (Fig 6E–6J).

### 2.7 Knockdown of circRNA-407 suppressed ovarian development

**Fig 7:**
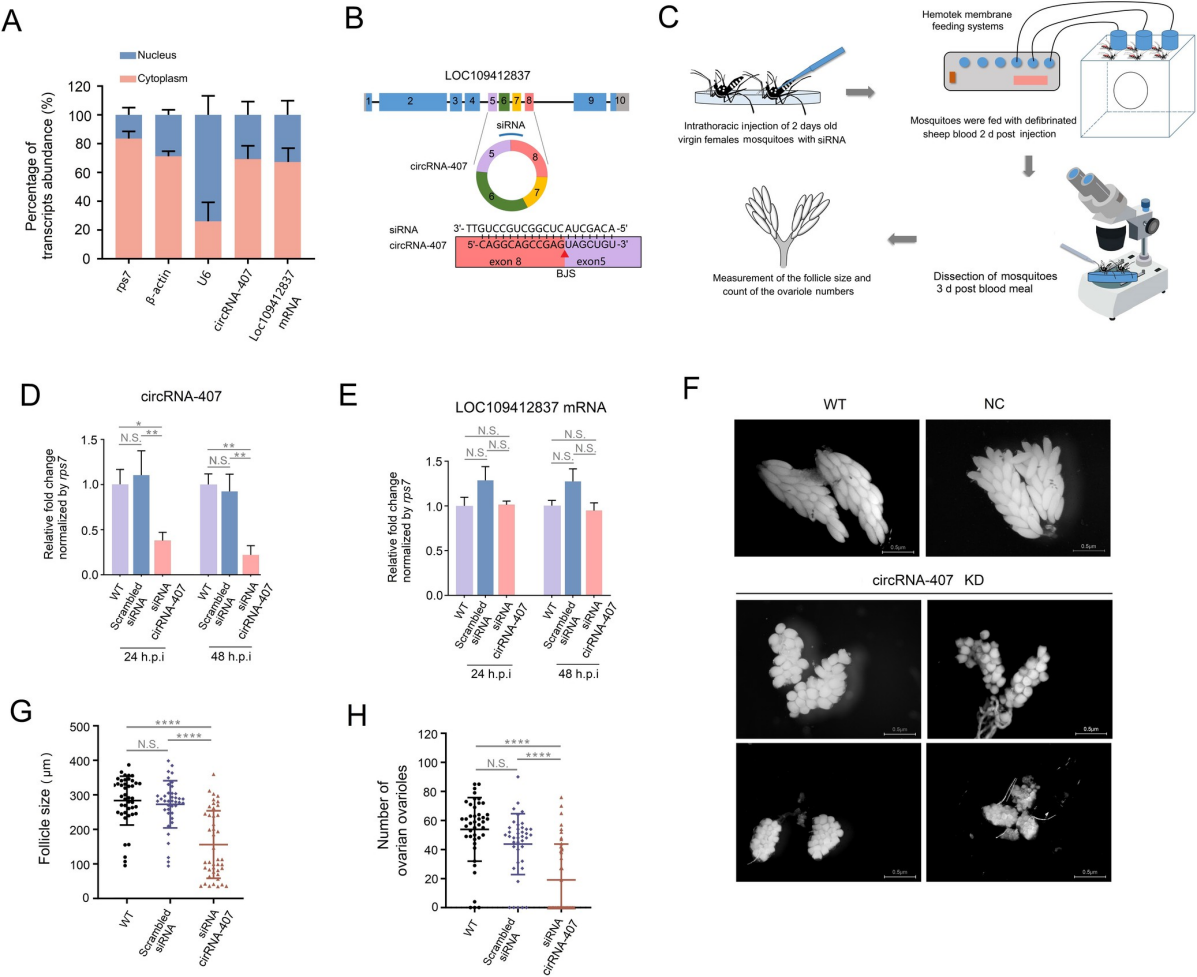
Effect of circRNA-407 knockdown on ovarian development in adult female *Ae*. *albopictus*. **(A)** The relative expression quantities of circRNA-407 in the cell nucleus and cytoplasm fractions of adult females (n = 15 females per replicate) were detected by qRT-PCR. *AalrpS7* and *β-actin* mRNA served as markers of cytoplasmic fractions, whereas *U6* served as a marker of nuclear fractions. **(B)** Illustration of siRNA design strategy for circRNA-407 specific knockdown. **(C)** Workflow diagram of the general processing analysis of effect of circRNA-407 knockdown on ovarian development. **(D)** and **(E)** qRT-PCR determined the relative expression of circRNA-407 (D) or its host genes *LOC1094128437* mRNA (E) in siRNA treatment groups compared with siRNA-NC and WT groups. Relative expression in WT groups was set as 1. qRT-PCR reactions were performed triplicates with three biological replicates (n = 15 females per replicate). **(F)** Representative images of ovaries dissected from circRNA-407 knockdown, siRNA-NC and WT female mosquitoes at 48 h PBM. All the images were taken with a Nikon SMZ1000 stereomicroscope with DIGITAL SIGHT DS-U3 (Scale bar: 0.5 μm). **(G)** and **(H)** Average follicle size (length of the long axis) (E) of ovaries isolated from female mosquitoes and number of developing follicles (F) per female individual in circRNA-407 knockdown (n = 43), NC (n = 40) and WT (n = 43) groups. All data was presented as means ±SEM and one-way ANOVA was used to compare the means among different groups. *p < 0.05; **p < 0.01; ****p < 0.0001; N.S., no significance.

Considering its distinct spatial expression in fat bodies and upregulation post blood meal in female fat bodies, we further explored the potential function of circRNA-407 in fat bodies during ovarian development. We first determined the subcellular localization of circRNA-407 in fat body cells. Briefly, the fat body of female mosquitoes was dissected, and nuclear and cytoplasmic fraction separation was performed as described in the methods. Levels of circRNA-407, *LOC109412837* (host gene of circRNA-407) mRNA, *β-actin*, *rps7* and *U6* snRNA in purified nuclear and cytoplasm fractions were measured by qRT-PCR. As a result, 69.33% of circRNA-407 and 96.53% of its host mRNA were detected in the cytoplasm fraction, which indicated that both circRNA-407 and its host mRNA are localized predominantly in the cytoplasm of adult female cells (Fig 7A).

Since circRNA-407 was prominently expressed in the cytoplasm of adult fat bodies, we next sought to determine the function of circRNA-407 in association with vitellogenesis and mainly focused on ovarian development by using siRNA-mediated circRNA interference. The siRNA was designed to target the specific BSJ of circRNA (sense 5’-CAGGCAGCCGAGUAGCUGUTT-3’, antisense 5’-ACAGCUACUCGGCUGCCUGTT-3’), and a scrambled siRNA was used as a negative control (siRNA-NC) (Fig 7B). The overall processing workflow is depicted in Fig 7C. Briefly, 2 days post emergence virgin adult females were thoracic injected with siRNAs using a nanoinjector. Forty-eight hours post injection, mosquitoes were fed on defibrinated sheep blood, and only freshly fed mosquitoes with a fully engorged abdomen with bright red blood were picked out and dissected 48 h PBM per group to analyze the effect of RNAi on ovarian development. First, the knockdown efficiency of the siRNA was assessed by qRT-PCR. The results showed that siRNA targeting the BSJ of circRNA-407 significantly reduced its expression in whole body of the females to 34.39% and 23.82% 24 h and 48 h post injection, respectively, compared with the siRNA-NC groups (Fig 7D). To further confirm the specificity of the knockdown efficiency, the expression level of the linear mRNAs generated from the host gene *LOC109412837* was also measured by qRT-PCR. As expected, treatment with BSJ-siRNA did not impact the expression of the host gene mRNAs, suggesting that the knockdown is specific to circular circRNA-407 (Fig 7E). After determining the knockdown efficiency of the siRNA, the siRNA-treated mosquitoes were given a blood meal and dissected at 48 h PBM for phenotypic manifestation observation. Interestingly, compared with the wild-type (WT) and siRNA-NC groups, we observed that circRNA-407 knockdown females failed to develop fully vitellogenic ovaries (Fig 7F). We then measured the average follicle size (length of the long axis) and counted the developing follicle numbers per mosquito. Compared with the WT (283.39 ± 70.67μm) and siRNA-NC (272.87 ± 68.32μm) groups, ovarian follicle growth in the circRNA-407 knockdown groups was inhibited, with an average primary follicle length of 160.28 ± 97.86 μm. In addition, knockdown of circRNA-407 caused a significant decrease in the number of developing follicles (20.50 ± 24.67, n = 43) compared with the WT group (53.88 ± 21.98, n = 43) (*p* < 0.001) and siRNA-NC group (43.73 ± 20.98, n = 40) (*p* < 0.001) (Fig 7G and 7H).

### 2.8 Overexpression of circRNA and RfxCas13d-mediated circRNA Knockdown in C6/36 Cells

**Fig 8:**
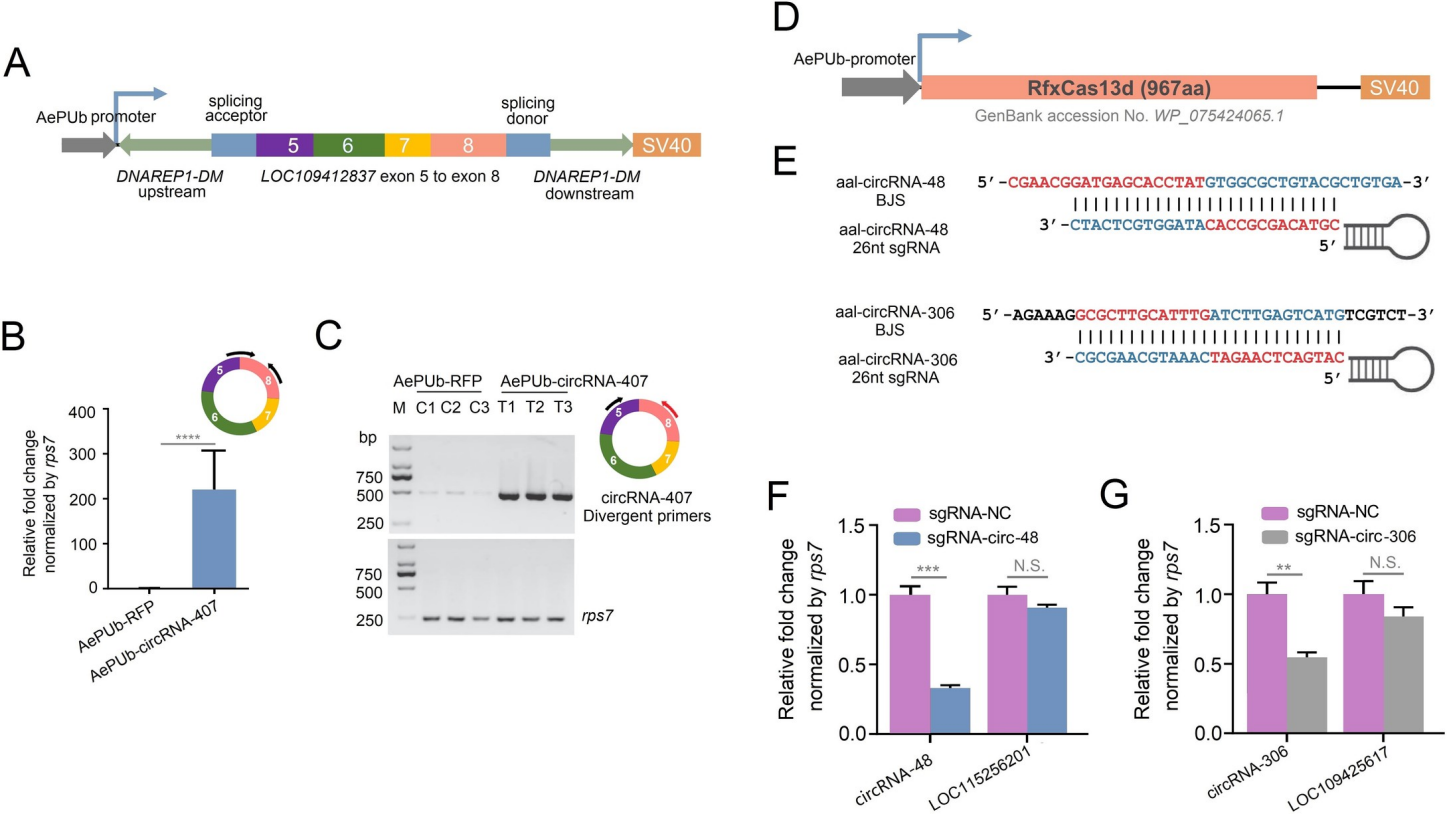
Overexpression of circRNA and RfxCas13d-mediated circRNA knockdown. **(A)** Schematic of circRNA-407 overexpression plasmid. Expression of circRNA-407 was driven by AePUb promoter and circularization of *LOC109412837* exon 5 to exon 8 was facilitated by *Drosophila DNAREP1-DM* flanking intron. **(B)** Overexpression of circRNA-407 in C6/36 was confirmed by qRT-PCR compared with C6/36 transfected with AePUb-RFP plasmid. Colored circle represented circRNA-407 and the black arrow pairs indicated BSJ-overlapping primers used for qRT-PCR. Relative expression of circRNA-407 in AePUb-RFP transfected cells was set as 1. **(C)** Agarose gel electrophoresis showing overexpression of circRNA-407 in C6/36 (T1, T2 and T3) compared with cells transfected with AePUb-RFP (C1, C2 and C3). Expected bands were amplified by BSJ-spanning primers as the arrows indicated. *AalrpS7* was used as an endogenous control. **(D)** Schematic of RfxCas13d expression plasmid. Transcription of RfxCas13d was driven by AePUb promotor and SV40 poly(A) served as signal of transcription termination. **(E)** Illustration of sgRNA design of RfxCas13d mediated circRNA knockdown. 26 nt sgRNAs were designed according to the BSJ of circRNA-48 and circRNA-306 respectively. **(F)** and **(G)** Relative expression of circRNA-48 (F) or circRNA-306 (G) and their linear host gene mRNA in C6/36 co-transfected with AePUb-RfxCas13d and BSJ specific 26nt sgRNA was quantified by qRT-PCR comparing with cells co-transfected with sgRNA-NC. Relative expression of circRNA and mRNA in sgRNA-NC transfected cells was set as 1. All qRT-PCR reactions were performed triplicates with three biological replicates and data was shown as means ±SEM. Student’s t-test was used to compare the means between two groups.**p < 0.01; ***p < 0.001; ****p < 0.0001; N.S., no significance.

To better explore and understand the mechanism of circRNA-407 in controlling ovarian development in female mosquitoes, we tried to construct effective tools for studying circRNAs in mosquito species. Long complementary flanking introns have been reported to facilitate the biogenesis of circRNAs [33–34], and many studies have utilized this strategy to successfully overexpress circRNAs *in vivo* and *in vitro* [35–36]. Here, we referred to the overexpression of circRNAs in fruit flies with slight modification to construct a mosquito applicable circRNA overexpression vector as described in Materials and Methods [37]. Briefly, the *D*. *melanogaster Laccase2* flanking introns (*DNAREP1_DM* repeats) were linked between the specific circularized exon along with the splicing acceptor and splicing donor. The entire sequence was then cloned downstream of the *Ae*. *aegypti polyubiquitin* (AePUb) promoter to replace the RFP gene of the AePUb-RFP plasmid. According to this method, we constructed the AePUb-circRNA-407 plasmid (Fig 8A). *Ae*. *albopictus* C6/36 cells transfected with the AePub-circRNA-407 plasmid successfully overexpressed circRNA-407 up to 200 times. (Fig 8B). Furthermore, overexpression of circRNA-407 was also confirmed by agarose gel electrophoresis, and the BSJ site was confirmed by Sanger sequencing of PCR products (Fig 8C), indicating that the expected BSJ is correctly circularized. Later, we tried to explore whether this method could also overexpress other circRNAs *in vitro*. Here, exon 2 of the *Aaldsx* gene, which also generated a circRNA in our circRNA-seq data (circDSX), was cloned into our AePUb-circRNA vector (S2A Fig). As expected, AePUb-circDSX successfully overexpressed circDSX up to 5,000 times (S2B and S2C Fig), and the BSJ sequence was further confirmed by Sanger sequencing, again verifying the efficiency of our circRNA expression vector in the C6/36 cells. To further explore whether this circRNA overexpression strategy could also function *in vivo*, the AePub-circRNA-407 plasmid was thoracic injected into the female mosquitoes. However, we could not observe an upregulation of circRNA-407 after injection of AePub-circRNA-407 plasmid in female mosquitoes. The circularization of circRNA seemed to be limited by the length of the linear exon but not the plasmid, because we successfully overexpressed an artificial circRNA (364 nt) in female *Ae*. *aegypti* (S6A–S6C Fig). Therefore, this circRNA overexpression strategy still need further modification.

Although siRNA-mediated circRNA knockdown has been proven to be sufficient and an easy way to study loss of function in circRNAs [38], the design of circRNA-specific siRNAs is limited to their BSJ sites [39]. In some cases, it is not even possible to design effective siRNAs [23]. A recent study proved that the CRISPR–RfxCas13d system is a useful tool for knockdown of circRNAs without affecting their host gene mRNAs in mammals [40]. In addition, CRISPR-Cas13a system-mediated gene silencing has been proven to be effective in mosquitoes [41]. Here, we wondered whether CRISPR–RfxCas13d system-mediated circRNA knockdown could also work in the mosquito system. Therefore, the codon-optimized RfxCas13d coding sequence (NCBI accession No. *WP_075424065*.*1*) was synthesized and cloned into the AePUb-RFP plasmid, named AePUb-RfxCas13d (Fig 8D). We generated two sgRNAs with spacers of 26 nucleotides complementary to the BSJ of circRNA-48 (sgRNA-circ-48) and circRNA-306 (sgRNA-circ-306) (Figs 8E, S5C and S5D), and a sgRNA with the same length targeting a human circRNA, has-circPOLR2A, was used as a negative control (sgRNA-NC). As shown in Fig 8F and 8G, cotransfection of AePUb-RfxCas13d and specific BSJ-sgRNA effectively decreased the expression of circRNA-48 and circRNA-306 by more than 50% but did not affect their linear host mRNAs compared with sgRNA-NC groups. Taken together, we successfully constructed effective and applicable methods to overexpress and knockdown circRNAs in C6/36 cells.

### 2.9 Aal-circRNA-407 could not be translated but acted as a miRNA sponge

With the help of constructing effective tools for studying circRNAs in C6/36 cels, we continued to explore the mechanism of aal-circRNA-407 in regulating ovarian development in adult females. Many functional circRNAs have been verified to be capable of translating into peptides or proteins and directly executing physiological regulation [42]. Although the translation of circRNAs has been shown to be regulated in different ways, translatable circRNAs are supposed to possess at least one open reading frame (ORF) in their circularized nucleotide sequence [43]. Therefore, we first analyzed the sequence of circRNA-407 and determined whether it possessed an ORF. Online ORF prediction showed that circRNA-407 harbored an ATG start codon in-frame with the stop codon directly located after BSJ (S3A Fig). To verify the translation ability of circRNA-407, two vectors were established based on the AePUb-circRNA-407 plasmid. Sequence of V5 tag (GKPIPNPLLGLDST), which derived from the P and V protein of the simian virus 5 (SV5, a paramyxovirus), amino acid residues 95 to 108 of RNA polymerase alpha subunit [44], was separated and added in reverse to both sides of circRNA-407 before the stop codon of the putative ORF (AePub-5-circ407-V). A vector expressing the linearized ORF with a V5 tag was constructed as a positive control (AePub-linear407-V5) (S3B Fig). These two plasmids were transfected into C6/36 cells. Circularization of AePub-5-circ407-V was verified by RT-PCR using divergent primers, and the junction site was correctly spliced by Sanger sequencing of the PCR products (S3C Fig). Four days post transfection, cells were collected to detect the potential translated products. The antiV5 antibody detected only an approximately 31.40 kDa protein in the positive control, AePub-linear407-V5 transfected cells, indicating that circRNA-407 may not be involved in ovarian development through coding peptides or proteins (S3D Fig).

**Fig 9:**
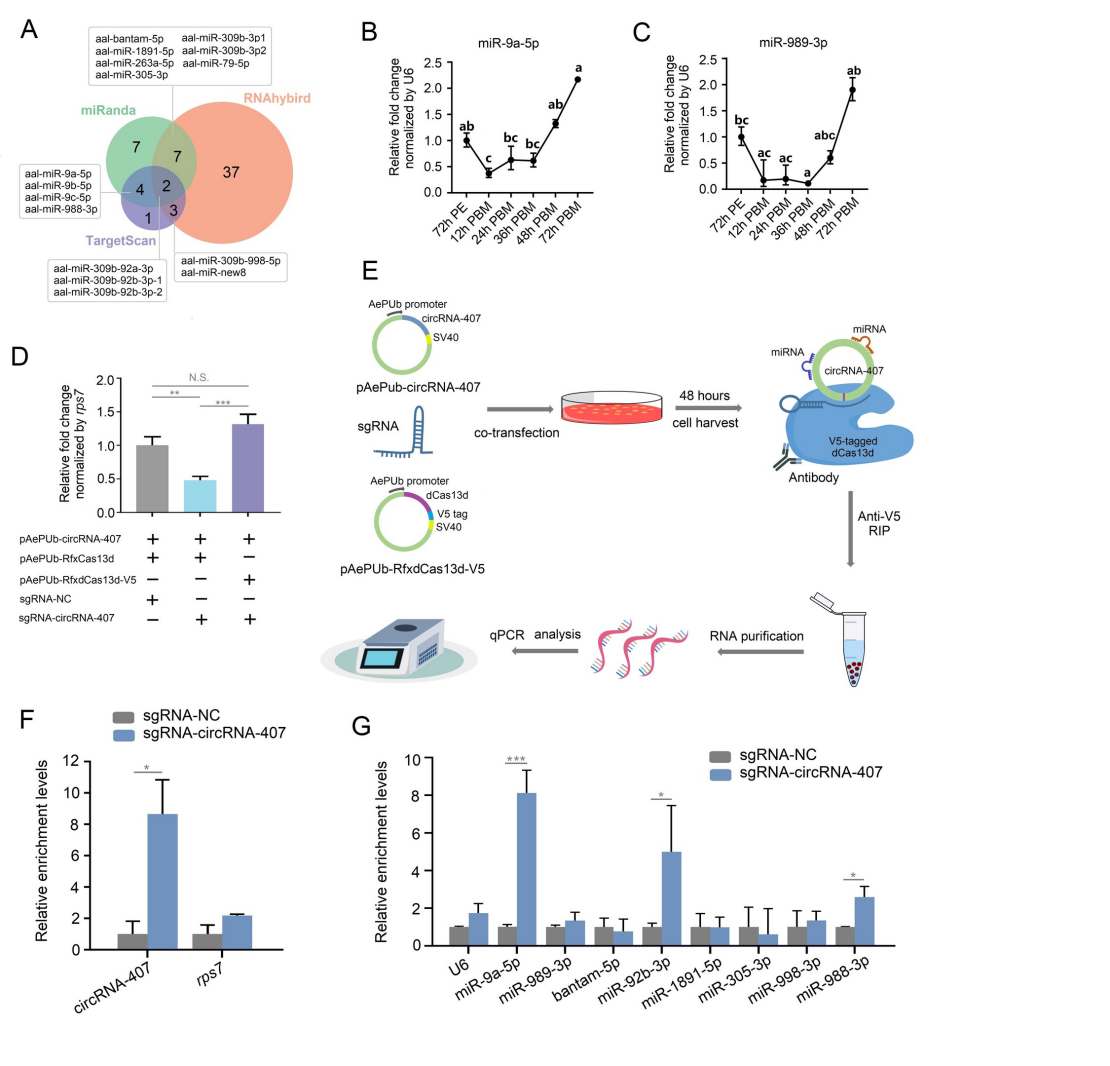
CircRNA-407 could act as miRNA sponge. **(A)** Venn diagram showing the putative miRNAs interacting with circRNA-407 predicted by miRanda, RNAhybrid and TargetScan. **(B)** and **(C)** Dynamic expression levels of miR-9a-5p (B) or miR-989-3p (C) in fat body of female mosquitoes (n = 15 females per replicate) after a blood meal determined by qRT-PCR. Samples were collected at 72 h post emergence (72h PE), 12 h, 24 h, 36 h, 48 h and 72 h post blood meal (PMB). Relative expression of miRNA at 72 h PE was set as 1. **(D)** qRT-PCR analysis showed that pAePUb-dRfxCas13d-V5 failed to suppress overexpression of circRNA-407. **(E)** Workflow showing the Dead-RfxCas13d-sgRNA-mediated RNA immunoprecipitation. **(F)** Dead-RfxCas13d-sgRNA-mediated RNA immunoprecipitation were performed using a 26 nt sgRNA targeting BSJ of circRNA-407 (sgRNA-circRNA-407) or a human circRNA hsa-circPOLR2A (sgRNA-NC). Relative enrichment level of circRNA-407 and *AalrpS7* in sgRNA-circRNA-407 groups was evaluated by qRT-PCR compared with sgRNA-NC groups. **(G)** The relative enrichment level of potential target miRNA of circRNA-407 in sgRNA-circRNA-407 groups was assessed by qRT-PCR compared with sgRNA-NC groups. All qRT-PCR reactions were performed triplicates with three biological replicates and data was shown as means ±SEM. Student’s t-test was used to compare the means between two groups and one-way ANOVA was used to compare the means among different groups. *p < 0.05; **p < 0.01; ***p < 0.001; ****p < 0.0001; N.S., no significance. Different letters above the bars denote statistical significance at *p* < 0.05.

Next, we conjectured whether circRNA-407 could function as a miRNA sponge, since it was among the most abundant circRNAs in our circRNA-seq data and was highly expressed in the female fat body, and various studies have revealed that miRNAs participate in ovarian development or metabolism of the blood meal by regulating their target mRNAs [45–46]. Three software programs, RNAhybrid [47], TargetScan [48] and miRanda [49], were used to predict the potential miRNA binding sites on circRNA-407. The miRNAs that were predicted by at least two software programs are listed in Fig 9A. Among these miRNAs, we focused mainly on miRNAs that have been reported to be involved in nutrition metabolism, ovarian development or reproduction. The dynamic expression levels of several predicted miRNAs in fat bodies during oogenesis were determined by qRT-PCR. Here, we noticed that two miRNAs, miR-9a-5p and miR-989-3p, displayed trends opposite to circRNA-407 in the fat body after a blood meal. The expression of both miRNAs decreased after a blood meal and remained at a low level until 36 h PBM and increased when circRNA-407 started to decrease at 48 h PBM (Fig 9B and 9C), while other miRNAs also showed a dynamic change during oogenesis (S4A–S4E Fig). To screen out functional miRNAs that may interact with circRNA-407, we created a novel method to capture circRNAs and their interacting molecules since we failed to enrich target circRNA using the traditional circRNA-specific biotin-labeled probe mediated RNA-pulldown assay. Alternatively, we performed dead-RfxCas13d-sgRNA-mediated RNA immunoprecipitation (RIP) as described in the Materials and Methods. Briefly, we mutated the active sites of RfxCas13d, which resulted in the loss of its RNA-cleaving function but retained its crRNA binding and target RNA binding activities, dead-RfxCas13d (AePUb-dRfxCa13d-V5). As shown in Fig 9D, compared with the AePUb-circRNA-407 and AePUb-RfxCas13d cotransfection groups, cotransfection of AePUb-circRNA-407 and AePUb-dRfxCas13-V5 failed to inhibit the overexpression of circRNA-407. Western blot using the antiV5 antibody further confirmed the expression of mutated RfxCas13d protein (S4F Fig). Therefore, we tried to use this strategy to perform the RIP assay to indirectly capture circRNA-407 and its interacting miRNAs (Fig 9E). Because the two miRNAs of interest, miR-9a-5p and miR-989-3p, were not expressed in C6/36 cells, we cotransfected C6/36 with AePUb-circRNA-407, AePUb-deadCas13d-V5, miR-9a mimics and miR-989 mimics, along with sgRNA that targeted BSJ of circRNA-407 (sgRNA-407) or human circPOLR2A (sgRNA-NC). The RIP assay was performed 72 h post transfection, and total RNA and proteins were extracted from the immunoprecipitation complex for subsequent detection of different molecules. Western blot analysis indicated that the RIP assay efficiently captured the deadRfxCas13d-V5 complex (S4G Fig). As shown in Fig 9F, qRT-PCR demonstrated that circRNA-407, but not *AalRps7*, was enriched in the dead-Cas13d-V5-sgRNA-407-mediated RIP complex compared with the sgRNA-NC groups, suggesting that we successfully captured circRNA-407 using the novel indirect method described above. Next, enrichment of the candidate miRNAs predicted by the bioinformatics in sgRNA-407 groups compared with sgRNA-NC was quantified by qRT-PCR. As a result, miR-9a-5p, but not miR-989-3p, which was co-overexpressed with miR-9a-5p, was significantly enriched in the sgRNA-407 groups. Meanwhile, miR-92-3p and miR-988-3p were also enriched in the sgRNA-407 groups, whereas other predicted miRNAs or *U6* snRNA showed no difference in enrichment between the sgRNA-407 and sgRNA-NC groups (Fig 9G).

### 2.10 Aal-circRNA-407 sponged miR-9a-5p to regulate ovarian development

**Fig 10:**
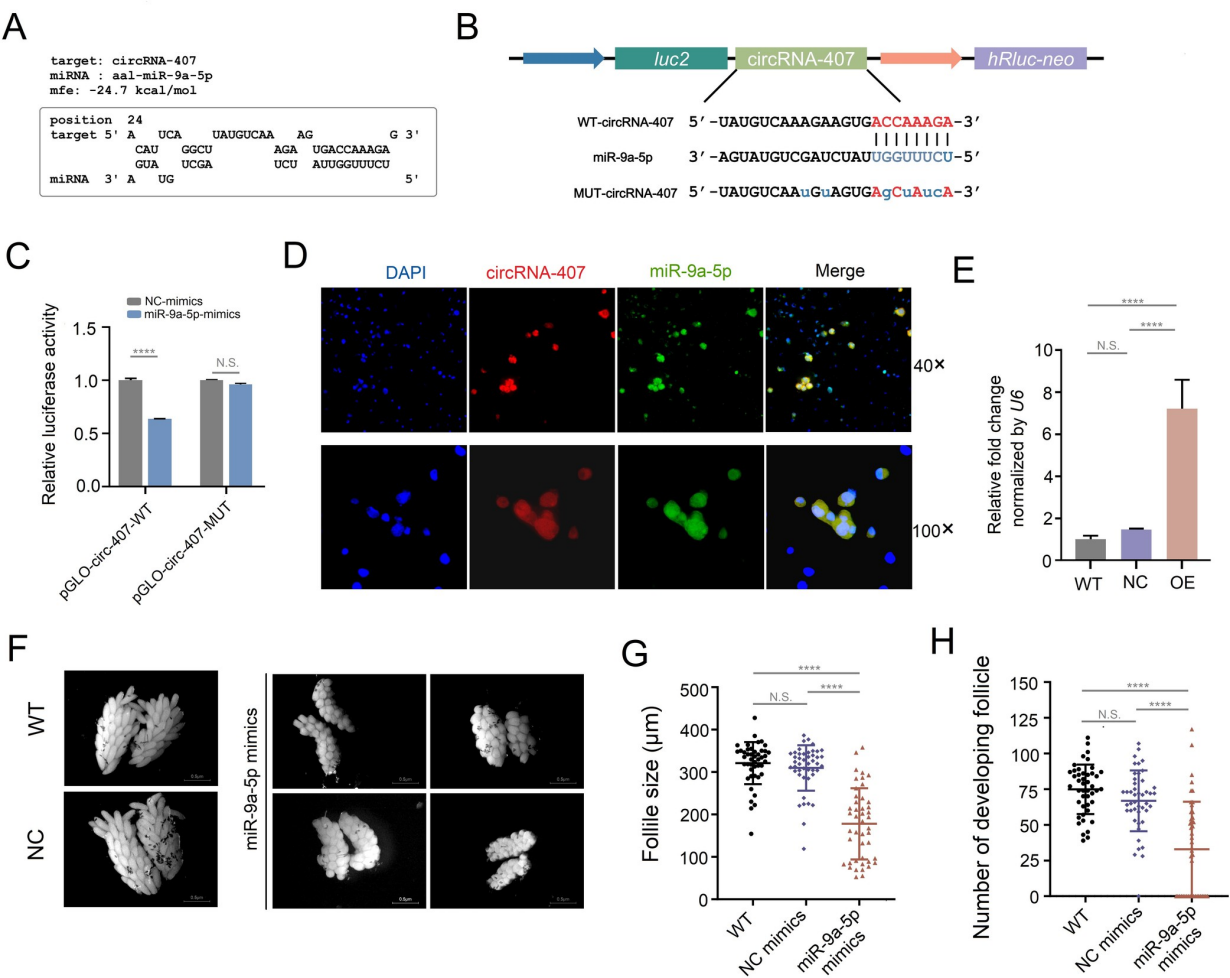
CircRNA-407 sponged miR-9a-5p to regulate ovarian development. **(A)** The putative miR-9a-5p binding site in circRNA-407 was predicted by RNAhybrid. **(B)** Schematic diagram of dual-luciferase reporter gene structure based on pmir-GLO plasmid. The putative binding sites (WT-circRNA-407) and the mutant version (MUT-circRNA-407) for circRNA-407 was presented. **(C)** Relative luciferase activity was determined 48 h after co-transfecting HEK293T cells with miR-9a-5p/NC-mimics (n = 3) and circRNA-407-WT/MUT (n = 3). Relative luciferase activity in NC-mimics groups was set as 1. **(D)** FISH assay analysis for the co-localization of miR-9a-5p and circRNA-407 in C6/36 cells. Nuclei was counterstained with DAPI (blue), probes for circRNA-407 was cy3-labeled (red) and probes for miR-9a-5p was FAM-labeled (green). **(E)** Relative expression level of miR-9a-5p in female mosquitoes upon thoracic injection of miR-9a-5p mimics (OE), negative control mimics (NC) and wild type blank control (WT) determined by qRT-PCR. qRT-PCR reactions were performed triplicates with three biological replicates (n = 15 females per replicate). **(F)** Representative images of ovaries dissected from miR-9a-5p mimics treated, NC-mimics and WT female mosquitoes at 48 h PBM. All the images were taken with a Nikon SMZ1000 stereomicroscope with DIGITAL SIGHT DS-U3 (Scale bar: 0.5 μm). **(G)** and **(H)** Average follicle size (length of the long axis) (G) of ovaries isolated from female mosquitoes and number of developing follicles (H) per female individual in miR-9a-5p mimics treated (n = 45) NC-mimics (n = 45) and WT (n = 45) mosquitoes. All data was shown as mean ± SEM. The Student’s t-test was used to compare the means between two groups and one-way ANOVA was used to compare the means among different groups. ****p < 0.0001; N.S., no significance.

Given that the expression of miR-9a-5p in the female fat body displayed an inverse dynamic change compared with circRNA-407 during ovarian development (Fig 9B) and that our deadCas13-sgRNA-mediated RIP successfully enriched circRNA-407 together with miR-9a-5p (Fig 9F and 9G), we further tried to validate the interaction between circRNA-407 and miR-9a-5p. Bioinformatics indicated that there were binding sites for miR-9a-5p on circRNA-407 (Fig 10A). To confirm the bioinformatics prediction, we conducted a dual luciferase reporter assay in HEK293T cells. A 700-bp sequence of circRNA-407 harboring the putative miR-9a-5p binding site was cloned into the 3’UTR of the *firefly luciferase* gene (*luc2*) in the pmirGLO plasmid (pGLO-circ407-WT), and the miR-9a-5p binding site (pGLO-circ407-MUT) was mutated (Figs 10B and S5A). Cotransfection of pGLO-circ407-WT and miR-9a-5p mimics resulted in a significant reduction in the relative luciferase activity (Fig 10C), while there was no significant difference between the miR-9a-5p mimics and NC mimics in the relative luciferase activity of the pGLO-circ407-MUT groups. Furthermore, fluorescence in situ hybridization (FISH) showed that the cy3-labeled circRNA-407 probe colocalized with the FAM-labeled miR-9a-5p probe predominantly in the cytoplasm in C6/36 cells (Fig 10D). Taken together, these results indicated that circRNA-407 can bind miR-9a-5p.

To further confirm whether circRNA-407 regulates ovarian development by sponging miR-9a-5p in the fat body of female mosquitoes, we tried to explore whether overexpression of miR-9a-5p in adult females could result in a similar phenotypic change that was observed in knockdown of circRNA-407 during oogenesis. Virgin female mosquitoes were thoracically injected with miR-9a-5p mimics (Fig 10E) and were given a blood meal 48 h post injection. Phenotypic observation was executed as described above. As expected, overexpression of miR-9a-5p also resulted in a deficiency of ovarian development that resembles the knockdown of circRNA-407 (Fig 10F, 10G and 10H). Collectively, the above results suggested that circRNA-407 is involved in ovarian development by acting as a sponge for miR-9a-5p in the fat body of female mosquitoes during oogenesis.

### 2.11 Aal-circRNA-407 regulated ovarian development through the circRNA-407/miR-9a-5p-*Foxl* axis in the fat body of female *Ae*. *albopictus*

**Fig 11:**
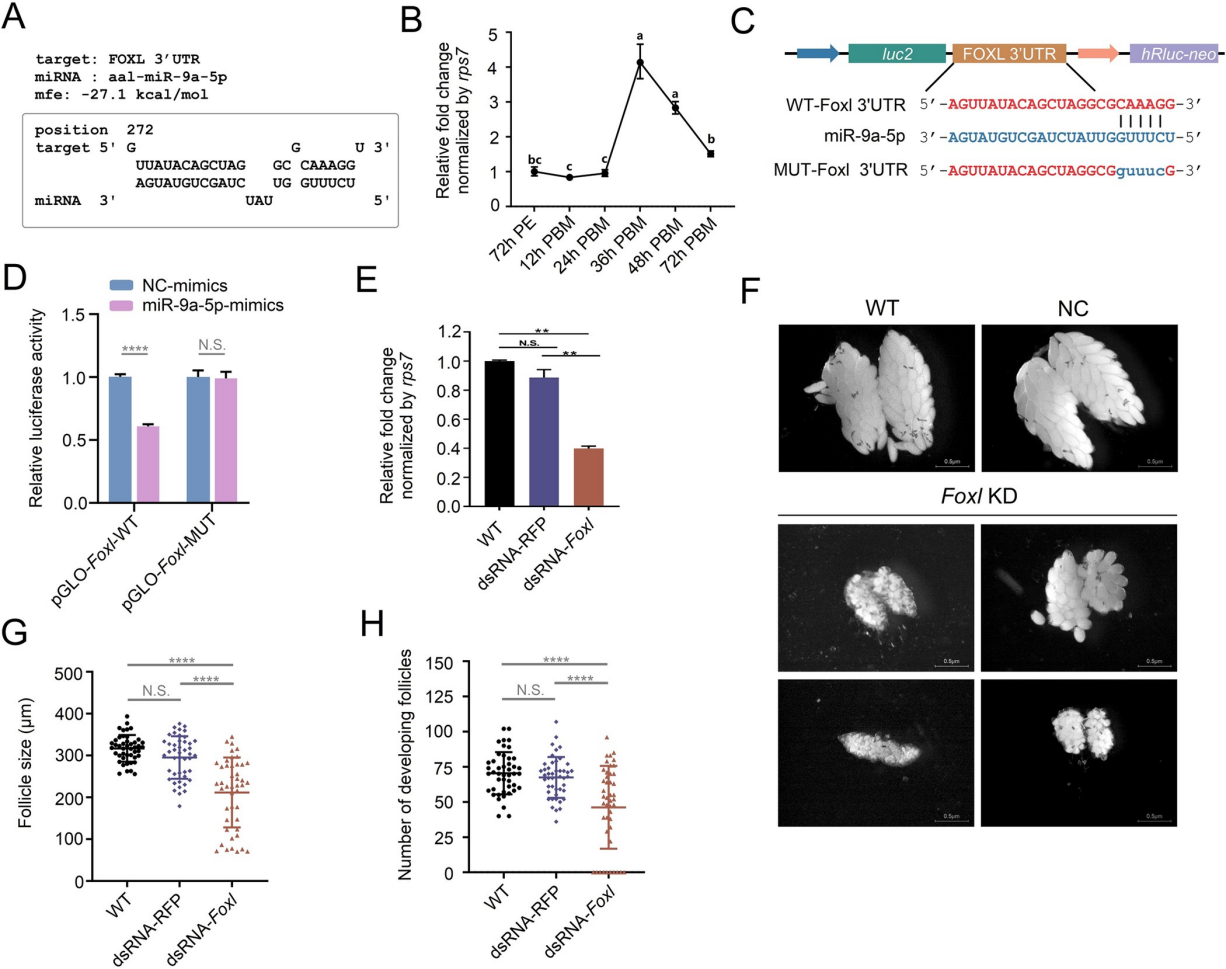
CircRNA-407 regulated ovarian development via miR-9a/*Foxl* axis. **(A)** The putative miR-9a-5p binding site in 3’UTR of *FOXL* was predicted by RNAhybrid. **(B)** Dynamic expression levels of *Foxl* in fat body of female mosquitoes (n = 15 females per replicate) after a blood meal determined by qRT-PCR. Relative expression of *Foxl* at 72 h PE was set as 1. **(C)** Schematic diagram of dual-luciferase reporter gene structure based on pmir-GLO plasmid. The putative binding sites (WT-*Foxl* 3’UTR) and the mutant version (MUT-*Foxl* 3’UTR) for miR-9a-5p was presented. **(D)** Relative luciferase activity was determined 48 h after co-transfecting HEK293T cells with miR-9a-5p/NC-mimics (n = 3) and WT/MUT-*Foxl* (n = 3). Relative luciferase activity in NC-mimics groups was set as 1. **(E)** Relative expression of *Foxl* in *Foxl*-dsRNA injected groups (dsRNA-*Foxl*) compared with RFP-dsRNA injected control (dsRNA-RFP) and WT groups. The expression level of *Foxl* in WT groups was set as 1. **(F)** Representative images of ovaries dissected from *Foxl*-dsRNA treated (*Foxl* KD), RFP-dsRNA treated (NC) and WT female mosquitoes at 48 h PBM. All the images were taken with a Nikon SMZ1000 stereomicroscope with DIGITAL SIGHT DS-U3 (Scale bar: 0.5 μm). **(G)** and **(H)** Average follicle size (length of the long axis) (G) of ovaries isolated from female mosquitoes and number of developing follicles (H) per female individual in dsRNA-*Foxl* (n = 45), dsRNA-RFP (n = 45) and WT (n = 45) mosquitoes. All qRT-PCR reactions were performed triplicates with three biological replicates and data was shown as means ±SEM. Student’s t-test was used to compare the means between two groups and one-way ANOVA was used to compare the means among different groups. **p < 0.01; ****p < 0.0001; N.S., no significance. Different letters above the bars denote statistical significance at *p* < 0.05.

Next, to determine the potential target genes of miR-9a-5p, all *Ae*. *albopictus* transcripts from the NCBI database were used for bioinformatics analysis by RNAhybrid, TargetScan and miRanda as described above. As a result, 7,016 transcripts were commonly predicted by these three software programs to harbor binding sites for miR-9a-5p. We tried to identify the target genes of miR-9a-5p that were most likely to be related to ovarian development by reviewing the relevant literature mentioning about ovarian development and CLIP-seq data analysis in mosquitoes [45,50–52]. Here, we noticed that *forkhead box protein L*, *Foxl* (NCBI GenBank No. *LOC109621391)*, contained a putative binding site for miR-9a-5p in its 3’UTR (Fig 11A). The *forkhead box* genes encode a family of transcription factors, and interference with *Foxl* in *Ae*. *aegypti* has been reported to have a negative effect on vitellogenin gene expression and resulted in significantly fewer eggs laid [53], indicating its role in ovarian development. Expression profile analysis showed that the expression of *Foxl* mRNA in female *A*. *albopitcus* fat bodies was dramatically elevated at 36 h PBM and then declined gradually until 72 h PBM, displaying a strong positive correlation with circRNA-407 expression but an opposite trend to miR-9a-5p (Fig 11B). Therefore, we continued to assess whether *Foxl* was a real target gene of miR-9a-5p. The entire 3’UTR sequence of *Foxl* was cloned into the pmirGLO plasmid as described above (pGLO-Foxl-WT), and the putative binding site for miR-9a-5p was mutated (pGLO-*Foxl*-MUT) (Figs 11C and S5B). We then transfected the constructed pGLO-*Foxl*-WT or pGLO-*Foxl*-MUT reporter vectors into HEK293T cells combined with miR-9a-5p mimics or NC mimics, respectively. The results showed that the relative luciferase activity of the pGLO-*Foxl*-WT groups significantly decreased in the miR-9a-5p mimics groups compared with that in the NC mimics groups, but these effects disappeared in the pGLO-*Foxl*-MUT groups (Fig 11D). These findings suggested a direct interaction between miR-9a-5p and *Foxl* mRNAs.

Next, we investigated whether interference of *Foxl* could phenocopy the deficiency of ovarian development that was observed in both circRNA-407 knockdown and miR-9a-5p overexpression. DsRNA was designed to target the CDS region of *Foxl*, and the knockdown efficiency of dsRNA on *Foxl* mRNAs was verified by qRT-PCR (Fig 11E). Then, we thoracally injected the 72 h PE female mosquitoes with the *Foxl* dsRNA, and a blood meal was given 72 h post injection. Evaluation of ovarian development was carried out as described above. As expected, knockdown of *Foxl* analogously resulted in phenotypic defects of ovarian development (Fig 11F), with shortened follicle size (Fig 11G) and decreased number of developing follicles (Fig 11H). Taken together, the overall results suggested that an abundant circRNA in the female fat body, circRNA-407, played a role in regulating ovarian development via the miR-9a-5p/*Foxl* axis in a complex, multielement affecting and dynamically changing microenvironment.

## 3. Discussion

Given the recent progress in high-throughput sequencing technologies and bioinformatics analysis tools, a rapid increase in the biological function of circRNAs has been observed. Especially in humans, accumulating evidence shows that circRNAs play an indispensable regulatory role in gene expression, an essential role in the process of biological development, and participate in the occurrence and development of various human diseases, such as cancer, cardiovascular diseases, and neurodegenerative diseases [54]. In contrast, the biological roles of circRNAs in insects, especially in insect vectors, are still in their infancy and need further annotation. *Aedes albopictus* mosquitoes are the main vectors of highly pathogenic arboviruses for humans in China, such as Dengue virus *(*DENV*)* [55], Zika virus (ZIKV) [56] and Chikungunya virus (CHIKV) [57]. Although a class of endogenous ncRNAs, including miRNAs [51,58], piRNAs [59–60] and lncRNAs [61–62], which play crucial roles in various biological processes, including development, oogenesis, metabolism and interactions between pathogens and vectors, have been well characterized in mosquito species, the function of circRNAs remains unclear in mosquitoes. To date, only the circRNA expression profiles in *Culex pipiens pallens* have been outlined, which suggests that an overexpressed circRNA in deltamethrin-resistant strains might participate in deltamethrin resistance through the cpp-miR-1671/*CYP4G15* pathway [22].

In the present study, by performing rRNA-depleted, RNase R-treated circRNA sequencing of *Ae*. *albopictus*, we identified 5,420 and 7,016 circRNAs in adult females and males, respectively by detecting the presence of BSJ reads. Approximately 85% of the circRNAs originated from exons of protein coding genes. The length of circRNAs was concentrated between 200 and 1,000 nt. Some genes could produce more than one circRNA. By performing qRT-PCR, we also demonstrated that the highly abundant circRNA in adult mosquitoes showed tissue- and developmental stage-specific expression patterns. These were similar to cases for other insects. For example, circRNAs have been reported to increase with age, and 1,182 circRNAs identified in wild-type *Drosophila* showed tissue-specific expression [23]. A total of 3,916 circRNAs were identified in the silk gland of *Bombyx mori* and expressed in a tissue-specific manner [18]. Moreover, circRNAs exhibit evolutionary conservation among different species, including mammals [1], insects [19] and plants [63–64], and a *Drosophila* circRNA, circMbl, is even conserved in humans and mice [65]. Interestingly, the highly expressed circRNAs in *Drosophila* [66] were also present in our circRNA-seq data, and further RT-PCR amplification followed by Sanger sequencing using divergent primers confirmed the existence of the predicted BSJ (S5E–S5H Fig). The above characteristics suggested that circRNAs in *Ae*. *albopictus* were similar to those identified in other insect species [17–19,67], and some circRNAs were even conserved between mosquitoes and fruit flies.

Highly abundant circRNAs are expected to be involved in crucial biological pathways and cellular processes [28]. Therefore, four circRNA candidates that ranked in the top 10 most abundant circRNAs in both females and males were selected for experimental validation and a fat body enriched circRNA, aal-circRNA-407, was chosen for further functional analysis. Although some circRNAs are more ubiquitously expressed, most of them show a distinct tissue- and developmental stage-specific expression pattern [68]. Our results showed that, among the circRNA candidates, circRNA-407 displayed an obvious fat body-enriched expression pattern and blood feeding-dependent onset in the fat body. Furthermore, the expression level of circRNA-407 was significantly upregulated during the early vitellogenic period and subsequently steadily decreased, showing a temporal expression pattern similar to that of *VgA*.

The fat body is the center of metabolism of mosquitoes. In fat body cells, lipids, carbohydrates and proteins are the substrates and products of many pathways that can be used for energy production, accumulate as reserves, and mobilize at the specific stage of the life cycle, determining the reproduction and survival of an individual [30]. Our results showed that circRNA-407 presented a stable expression level during glycometabolism either in females or males, indicating the potential relationship between circRNA-407 and nutrition-dependent vitellogenic development in the fat body of adult females and posing the question of what the roles of circRNA-407 played during ovarian development. Knockdown of circRNA-407 led to distinct deficiency of ovarian development in females, further verifying our predictions that circRNA-407 plays important regulatory roles in ovarian development of female mosquitoes.

In recent years, multiple important functions of circRNAs in diverse vital biological processes have been reported in various vertebrates. The known molecular mechanisms include miRNA sponges, templates for protein translation, RBP binding molecules and transcriptional regulators [69]. In particular, circRNAs acting as miRNA sponges have been most frequently reported. However, to our knowledge, only three circRNA regulatory mechanisms have been clarified in insects. In *Drosophila*, circSfl can translate a truncated protein that shares the N-terminus with the full-length Sfl protein encoded by the host gene *sulfateless* (*Sfl*) and may interact with proteins similar to the full-length Sfl protein, eventually influencing *Drosophila* lifespan [23]. circMbl is generated by *Muscleblind* (*mbl*) in *Drosophila*, and circMbl performs a cis-regulatory function by competing with its linear host *mbl* mRNA [70]. In silkworm, circEgg not only sequesters the microRNA bmo-miR-3391–5p to promote its target gene *histone H3 lysine 9 acetylation* (*H3K9ac*) expression but also encodes a protein that functions and plays an important role in histone epigenetic modification [71]. The reason for the lack of further molecular mechanisms of circRNAs in insects is mainly because materials and methods studying circRNAs established in mammals cannot be directly applied in insects. Some established methods in insects are species-specific and even not applicable between different insect species.

Therefore, to better understand the molecular mechanism of circRNAs in mosquito species, a series of methods was established in the present study. First, overexpression of circRNAs in mosquitoes was performed according to the characteristics of long complementary flanking introns in other species. We analyzed the flanking 1,000 nt introns of the highly abundant circRNAs in *Ae*. *albopictus* genome but found no similar features. However, by utilizing the *D*. *melanogaster Laccase2* flanking introns (*DNAREP1_DM* repeats) [37], we successfully overexpressed specific circRNAs in C6/36 cells driven by the AePUb promoter. However, we failed to overexpress circRNA-407 (967 nt) *in vivo* but successfully overexpressed an artificial circRNA (364 nt) in female *Ae*. *aegypti*. Such a phenomenon has also been observed in fruit flies [37]. There is also one possibility that the circularization ability is restricted by the length of flanking introns. Therefore, the overexpression of specific circRNAs in mosquitoes still needs further modification and exploration. Next, traditional siRNA- or shRNA-based circRNA knockdown usually results in off-target effects on their host genes, and their design is limited to BSJs [40]. Since RfxCas13d-sgRNA-mediated circRNA knockdown has been well established and applied in mammals [39], we also tested the efficiency of this method in a mosquito system. As expected, the RfxCa13d-sgRNA system was also applicable in C6/36 cells, resulting in specific knockdown efficiency on circRNAs without affecting their host genes. One of the effective ways to study circRNA-miRNA or circRNA-protein interactions is MS2 bacteriophage coat protein (MS2-CP) circRNA pull-down [72]. In brief, an MS2-tag is incorporated into the circRNA that can be recognized by MS2-CP protein, by which the specific circRNAs along with heir interaction molecules can be captured by performing MS2-CP immunoprecipitation [73]. However, such a strategy changes the real nucleotide sequence of a circRNA and may result in potential off-target purification of linear mRNA counterparts [74]. In the present study, taking advantage that RfxCas13d-mediated circRNA knockdown was applicable in C6/36 cells and efficient in knocking down circRNAs, we tried to establish a novel method to indirectly pull down the circRNA complex by utilizing the crRNA binding and target RNA binding activities of RfxCas13d. Briefly, a dead RfxCas13d expressing vector was constructed by mutating the RNA-degrading active sites. The combination of dead-RfxCas13d and circRNA was mediated by BSJ-specific sgRNA, and the complex was pulled down by performing antiV5 immunoprecipitation. This method did not change the nucleotide sequence of a circRNA, and BSJ-specific sgRNA could avoid off-target capture of linear mRNA counterparts. Finally, the circRNA molecular function analysis platform around the mosquito circRNA was successfully established, which lays a necessary foundation for the functional exploration of mosquito circRNAs.

CircRNAs are not real noncoding RNAs since many circRNAs have been validated to translate and directly execute their cellular physiology function [42,75]. A circRNA is well known to be able to act as a ceRNA to sponge and inhibit the activity of miRNAs [10], which bind to the 3’UTR of mRNAs to suppress their translation and promote their degradation [76]. Although a putative ORF was detected in circRNA-407, which potentially encodes a protein of 279 aa in length (designated circRNA-407-279aa) with a predicted molecular weight of 31.40 kDa, our results validated that circRNA-407 could not be translated into peptides. Therefore, we tried to explore whether circRNA-407 acted as an miRNA sponge to regulate ovarian development. By performing bioinformatics prediction and dead-RfxCa13d-sgRNA-mediated circRNA immunoprecipitation, we found that miR-9a-5p was the circRNA-407 binding miRNA, which was later confirmed by luciferase reporter gene and FISH assays. In the fat body of adult females during oogenesis, miR-9a-5p decreased and remained at a low expression level before 36 h PBM but dramatically increased at 48 h PBM when circRNA-407 started to decline. From a functional study, we found that upregulation of miR-9a-5p resulted in significantly shortened follicle size and decreased number of developing follicles that resembled the phenotype observed in knockdown of circRNA-407. Therefore, we speculated that circRNA-407 may have a positive effect on ovarian development in the female fat body by inhibiting the activity of miR-9a-5p.

To explore the complete regulatory pathway, we first screened the potential target genes of miR-9a-5p by using bioinformatics analysis and referring to relevant literature [45,50–52]. The results of the dual-luciferase reporter assay confirmed that *Foxl* was a direct target of miR-9a-5p. Expression of *Foxl* displayed a similar but reverse trend during oogenesis in the female fat body, and knockdown of *Foxl* also resulted in deficiency of ovarian development.

Previous studies have also revealed that miR-9a-5p can regulate members of the Forkhead box (FOX) family in mice [77] and humans [78–79]. FOXL is a member of the FOX, which encodes a family of transcription factors. Members of the FOX family have been identified and conserved in eukaryotic organisms from yeast to humans and have been shown to play important roles in regulating the expression of genes involved in various biological processes, such as development, metabolism and aging [53,80]. Previous studies on fruit flies also stress the importance of the FOX family in regulating development and homeostasis in *Drosophila* [81–84]. In *Ae*. *aegypti* [53], 18 putative Fox transcription factors were identified, and six of them expressed in the fat body displayed dynamic expression profiles following a blood meal. Moreover, knockdown of *FoxN1*, *FoxN2*, *Foxl*, and *FoxO* results in a negative effect on amino acid-induced vitellogenin gene expression, and significantly fewer eggs are laid. Although it has been indicated that *Foxl* functions as a transcription factor regulating the vitellogenin gene (*Vg*), the exact role of *Foxl* in *Ae*. *albopictus* still deserves further exploration and confirmation.

In summary, our results showed that a fat body*-*enriched circular RNA, aal-circRNA-407, regulated ovarian development in female *Ae*. *albopictus* after a blood meal via the miR-9a-5p/*Foxl* axis. Our study is the first to report a functional circRNA in mosquitoes, expanding our current understanding of the important roles of circRNAs in biological processes in *Ae*. *albopictus*. Uncovering the biological functions of circRNAs in this species will provide alternative genetic control strategies for vector control. For example, targeting a circRNAs that are indispensable for reproduction or are necessary for virus replication in mosquitoes by using well-established gain-of-function or loss-of-function approaches will provide us more approaches to control mosquito population and related diseases.

## 4. Materials and methods

### 4.1 Mosquito rearing and cell maintenance

Two mosquito species were used in this study. The *Ae*. *albopictus* Foshan strain established from field-collected specimens in Foshan, Guangdong, P.R. China and has been established in the laboratory since 1981. The *Aedes aegypti* Haikou strain was collected from Haikou, Hainan, P.R. China and has been maintained in an insectary since 2016. All mosquitoes were reared in a climate-controlled insectary at 27 ± 1°C with a relative humidity of 70%-80% under a 12 L:12 D photoperiod. Mosquito larvae were reared in pans and fed on small turtle food (INCH-GOLD, Shenzhen, China). Adult mosquitoes were kept in 20 cm*30 cm*45 cm yarn cages and allowed access to a cotton wick soaked in 10% glucose as a carbohydrate source. Adult females were fed defibrinated sheep blood (Solarbio Life Sciences, Beijing, China) supplied through a Hemotek membrane feeding system (Hemotek Ltd., Blackburn, UK).

*Ae*. *albopictus* C6/36 cells (American Type Culture Collection (ATCC)(Cat# CRL-1660, RRID: CVCL_Z230) were grown at 28°C in Roswell Park Memorial Institute (RPMI) 1640 medium (Gibco, Grand Island, NY, USA) supplemented with 10% fetal bovine serum (FBS; Gibco, USA). HEK293T cells were cultured in Dulbecco’s modified Eagle’s medium (DMEM) (Gibco, USA) with 10% FBS (Gibco, USA) and incubated in 5% CO_2_ at 37°C.

### 4.2 rRNA-depleted circRNA sequencing

First, total RNA was extracted from 2-day-old virgin female and male *Ae*. *albopictus* using Takara RNAiso Plus according to the manufacturer’s instructions (TAKARA, Dalian, China) (three biological replicates, n = 30 per replicate). The concentration and quality of RNA were measured using a Nanodrop Spectrophotometer (Allsheng, Hangzhou, China) and a Bioanalyzer 2100 system (Agilent Technologies, Santa Clara, CA, USA). RNA integrity was checked using a 1% agarose gel. RNA samples with an RNA integrity number (RIN) value of at least 7.0 or higher were used for further processing. After quality control, an RNA-seq library was prepared with approximately 2 μg of total RNA using a KAPA RNA HyperPrep Kit with RiboErase (HMR) for Illumina (Kapa Biosystems, Inc., Woburn, MA, USA). Briefly, DNA oligos were hybridized to rRNA and digested using RNase H treatment. The rRNA-depleted RNA was further treated with RNase R (Geneseed, Guangzhou, China) to remove the linear RNA and purified with VAHTS RNA Clean Beads (Vazyme, Nanjin, Chian). Next, RNA was fragmented into 200∼300 base pairs (bps), and strand-specific first-strand cDNA was generated using hexanucleotide random primers in the presence of actinomycin D to prevent spurious DNA-dependent synthesis. Subsequently, 2^nd^-strand cDNA was synthesized using dUTP instead of dTTP, and then A tailing and adapter ligation were performed with the purified cDNA. Uracil DNA glycosylase (UNG) was used to remove the cDNA strand containing dUTP. Finally, the purified, adapter-ligated DNA was amplified. The library quality and concentration were assessed by utilizing a DNA 1000 chip on an Agilent 2100 Bioanalyzer. Accurate quantification for sequencing applications was determined using the quantitative polymerase chain reaction (qPCR)-based KAPA Biosystems Library Quantification kit (Kapa Biosystems, Inc., Woburn, MA. USA). Each library was diluted to a final concentration of 10 nM and pooled equimolar prior to clustering. Paired-end (PE150) sequencing was performed on an Illumina HiSeq sequencer (Illumina, San Diego, CA, USA).

### 4.3 Bioinformatics analysis and circRNA prediction

The DCC pipeline [85] was used to predict circRNAs. Briefly, adapter contaminants, low-quality reads, reads containing poly N, and reads originating from rRNA were first removed from raw reads. All clean reads were aligned to the reference genome (*Aalbo_primary*.*1*) using STAR (version 2.5.2b) [86], and only fusion reads with no more than two mismatches were retained for further analysis. Then, the output files from STAR, chimeric.out.junction, were used for circRNA annotation with digital content creation (DCC) (https://github.com/dieterich-lab/DCC). Predicted circRNAs from DCC were filtered with at least one junction read in at least one group. Based on their genomic origins, circRNAs are classified into three major types: exonic, intronic, and intergenic circRNAs.

### 4.4 Differential expression analysis

For quantification of circRNAs, the numbers of back-spliced junction (BSJ) reads of each circRNA were normalized by CPM (counts of exon model per million mapped reads) values [27,87]. Those circRNAs for which the CPM values were less than 0.5 in half of the samples were filtered out. Differentially expressed circRNAs were estimated using the edgeR package (version 3.12.1) [88]. CircRNAs with |log_2_ fold change (log_2_FC)| > 1 and *p* value <0.05 were considered to be significantly differentially expressed between females and males.

### 4.5 circRNA PCR amplification and Sanger sequencing

To confirm the circRNAs predicted by circRNA sequencing, total RNA and genomic DNA were extracted using TRIzol reagent and a Universal Genomic DNA Extraction Kit (Takara, Dalian, China), respectively. Total RNA was treated with DNase I (Invitrogen, Waltham, MA, USA) and then reverse transcribed into cDNA with random primers by using the SuperScript IV First-Strand Synthesis System (Invitrogen, USA) according to the manufacturer’s instructions. A set of divergent and convergent primers (Sangon Biotech, Shanghai, China) were designed based on the sequences of candidate circRNAs (S1 Table). cDNA and gDNA were used as templates for each primer pair. PCR products were separated using gel electrophoresis in 1–2% agarose, purified with a QIAquick Gel Extraction Kit (Qiagen, Valencia, CA, USA) and submitted for Sanger sequencing on an Applied Biosystems 3130 sequencer (Waltham, MA,USA) for confirmation of the presence of BSJ.

### 4.6 Ribonuclease R (RNase R) treatment

Ten micrograms of total RNA was incubated with or without 40 U RNase R (Geenseed, Guangzhou, China) at 37°C for 20 min and then reverse transcribed to cDNA as described above. The tolerance of circRNA and mRNA to RNase R was compared by qRT-PCR.

### 4.7 Quantification of circRNAs, miRNAs and mRNA

Spatial-temporal patterns of candidate circRNA expression were quantified by qPCR analysis. Total RNA was extracted from different developmental stages and different adult tissues of *Ae*. *albopictus*, and first-strand cDNA was synthesized as described above. Then, cDNA amplification was performed using SuperReal PreMix Plus (SYBR Green) (Tiangen, Beijing, China). For miRNA quantification, reverse transcription was performed using the miRcute miRNA cDNA First-Strand Synthesis Kit (Tiangen, Beijing, China), and cDNA amplification was performed using miRcute miRNA SYBR qPCR Master Mix (Tiangen, Beijing, China). circRNA and mRNA transcript levels were normalized to *Ae*. *albopictus* ribosomal protein S7 gene (*AalrpS7*) expression, and miRNA transcript levels were normalized to *Ae*. *albopictus U6 spliceosomal RNA* (*U6*). Relative quantification of circRNA, mRNA and miRNA expression was analyzed using the comparative Ct (2^-ΔΔCt^) method. All reactions were performed in triplicate. The primer sequences (Sangon Biotech, Shanghai, China) are available in Additional file: S1 Table.

### 4.8 Isolation of nuclear and cytoplasmic fractions

For the assessment of the subcellular localization of circRNAs, adult female nuclei and cytoplasm were separated using a Cytoplasmic & Nuclear RNA Purification Kit (Norgen Biotek, Ontario, Canada) following the manufacturer’s instructions, and qRT–PCR analysis was performed as described above. *Ae*. *albopictus ribosomal protein S7* gene (*AalrpS7*) or *actin-5C* gene (*β-actin*) were used as indicators of cytoplasm enriched transcripts and *Ae*. *albopictus U6 spliceosomal RNA* (*U6*) was used as an indicator of nuclear-enriched transcripts.

### 4.9 Oligo and dsRNA treatment

All siRNAs, miRNA mimics and negative controls were designed and synthesized by GenePharma (Shanghai, China). dsRNAs were *in vitro* transcribed and purified using the T7 RiboMAX Express RNAi System (Promega, Madison, WI, USA). All oligo and dsRNA sequences are listed in S1 Table. For thoracic injection, 72 h post emergence (72h PE) female mosquitoes were anesthetized with ice and individually maintained on a glass plate over ice during the injection. Approximately 0.5 μL of solution (containing 100 μM/μL siRNA/miRNA mimics or 2 μg dsRNA dissolved with 1X injection buffer) [89] was injected laterally into the thorax using 15-μm needles with a CellTram 4r Oil microinjector (Eppendorf, Hamburg, Germany) under a stereomicroscope (piston position was set to 7–9 to generate optimal positive pressure) as described previously [90]. After injection, the female mosquitoes were immediately transferred to small plastic cups (900 mL, 11 cm top diameter) for recovery and fed with a 10% glucose solution through soaked cotton wicks. Whole body of the mosquitoes were collected at corresponding time points for gene expression analysis by qRT-PCR.

### 4.10 Ovarian dissection and phenotypic observation

72h PE female mosquitoes were treated with siRNAs, miRNA mimics or dsRNAs as described above. Injected mosquitoes were allowed recovery for 2 to 3 days before defibrinated sheep blood feeding. Only freshly blood-fed female mosquitoes with obviously engorged, bright red abdomens were selected for subsequent analysis. At 48 h post blood meal (PBM), the ovaries were dissected by tearing off the soft cuticle between the fifth and sixth abdominal sternites with a fine needle and then pulling off and placing the terminal segments in a drop of suitable mosquito saline buffer [91]. The ovaries were dissected, and two indicators were chosen to evaluate ovarian development: (1) the number of developing follicles, and (2) the follicle size per individual.

### 4.11 Construction of circRNA overexpression plasmid

The plasmid for overexpressing the circRNAs in *Ae*. *albopictus* was constructed according to the methods described in *Drosophilid* (37). Briefly, 422 nt upstream of the *Drosophila melanogaster Laccase2 DNAREP1_DM* repeat sequence followed by a splicing donor was linked to the 5’ end of the circularized exon, and a splicing acceptor followed by 540 nt downstream of the *DNAREP1_DM* repeat sequence was linked to the 3’ end of the circularized exon. This cassette was chemically synthesized (Sangon Biotech, Shanghai, China) and subcloned downstream of the *Ae*. *aegypti polyubiquitin* (AePUb) promoter between the *Not*I and *Nur*I restriction enzyme sites of AePUb-RFP (gift from Chun-Hong Chen). The *Ae*. *albopictus* C6/36 cells was transfected with the AePUb*-*circRNA expression plasmid by Lipofectamine 3000 Reagent (Invitrogen, USA) according to the manufacturer’s instructions, and transfection of the AePUb-RFP plasmid was used as a negative control. Total RNA was extracted 72 h post transfection and reverse transcribed into cDNA as described above. Overexpression and accurate BSJ circularization of circRNA was verified by qRT-PCR using BSJ-overlapping primers and Sanger sequencing of RT-PCR products using BSJ-spanning divergent primers.

### 4.12 RfxCas13d-sgRNA-mediated circRNA knockdown

The amino acid sequence of *Ruminococcus flavefaciens* type VI-D clustered regularly interspaced short palindromic repeats (CRISPR)-associated RNA-guided ribonuclease Cas13d (RfxCas13d) (NCBI accession NO. WP_075424065.1) was synthesized and then cloned downstream of the AePUb promoter (AePUb-RfxCas13d) after codon optimization of *Ae*. *aegypti*. sgRNAs were *in vitro* transcribed using the T7 RiboMAX Express Large Scale RNA Production System (Promega, USA). RfxCas13d-sgRNA-mediated circRNA knockdown was performed by cotransfecting C6/36 cells with AePUb-RfxCas13d and sgRNAs using Lipofectamine 3000 Reagent (Invitrogen, USA). Seventy-two hours post cotransfection, the cells were harvested, and the knockdown efficiency was determined by qRT-PCR as described above. All sgRNA sequences are available in Additional file: S1 Table.

### 4.13 Dead-RfxCas13d-sgRNA-mediated RNA immunoprecipitation

Three active sites of RfxCas13d were mutated (R239A, R858A and H863A), and a V5 tag (GKPIPNPLLGLDST) was infused into the C-terminus based on AePUb-RfxCas13d (AePUb-dCas13d-V5) using the Q5 Site-Directed Mutagenesis Kit (NEB, Ipswich, MA, USA). Mutated sites were confirmed by sequencing the plasmid. C6/36 cells were cotransfected with AePUb-dCas13d-V5, AePub-circRNA-407, miR-9a-5p mimics and miR-989-3p mimics accompanied by sgRNA-circRNA-407 or sgRNA-NC. RNA immunoprecipitation was performed according to a published method with modification [92]. Briefly, cells were harvested 72 h post transfection and lysed with radioimmunoprecipitation assay (RIPA) lysis buffer (50 mM Tris-HCl, pH 7.4, 150 mM NaCl, 0.25% deoxycholic acid, 1% NP-40, 1 mM ethylenediaminetetraacetic acid (EDTA) and 5% glycerol) supplemented with 40 U RNase Inhibitor (Promega, USA) and Protease Inhibitor (Beyotime, Shanghai, China). Two copies of 10% of the lysate were taken for subsequent protein extraction and RNA purification, and the remaining lysate was incubated with V5-Trap Magnetic Agarose (Proteintech, Rosemont, IL, USA) overnight at 4°C on rotation. Then, the beads were washed three times with RIPA buffer and resuspended in 500 μL RIPA buffer. Ten percent of the suspension was used for protein analysis, and the remaining suspension was used for RNA purification as described previously [92]. Equal amounts of RNA extracted from input and immunoprecipitations were then analyzed for enrichment of circRNAs and miRNAs using qRT-PCR as described above. Enrichment was normalized to 10% input using 2^−ΔCt^ and relative enrichment level to sgRNA-NC groups was calculated by 2^−ΔΔCt^.

### 4.14 Western blot

Western blot analysis was performed using standard procedures. Briefly, total proteins were extracted and boiled in 6× protein loading buffer for 10 min at 95°C, separated by 10% sodium dodecyl sufate (SDS)-polyacrylamide gels and transferred onto a polyvinylidene difluoride (PVDF) membrane (Millipore, Burlington, MA, USA). After blocking for 1 h with 5% bovine serum albumin (BSA), the membranes were incubated with monoclonal mouse antiV5 antibody (dilution 1:1000, Invitrogen, USA) and polyclonal mouse antiβ-actin antibody (dilution 1:1,000, Abcam, Cambridge, UK) overnight at 4°C. The next day, the membranes were detected with horseradish peroxidase (HRP)-conjugated mouse antigoat IgG (H+L) (dilution 1:5,000, ABclonal, Wuhan, China), followed by visualization by using enhanced chemiluminescent reagents.

### 4.15 Dual-luciferase assay

The pmirGLO Dual-Luciferase miRNA Target Expression Vector (Promega, USA) was used to construct the luciferase reporter vector. A 700 bp sequence of circRNA-407 containing the putative miR-9a-5p binding site and the full length of the 3’UTR of *Foxl* was amplified by RT-PCR and then inserted into the 3’UTR of the firefly luciferase gene (*luc2*) using In-Fusion HD Cloning Plus Kits (Takara, Shiga, Japan) according to the manufacturer’s instructions. The putative miRNA binding sites were mutated using the Q5 Site-Directed Mutagenesis Kit (NEB, USA). Wild-type or mutant luciferase reporter vectors with miR-9a-5p mimics or NC mimics were transfected into HEK293T cells. Two days later, the cells were lysed, and their luciferase activities were measured by using the Dual-Luciferase Reporter Assay System (Promega, USA) according to the manufacturer’s instructions.

### 4.16 Fluorescence in situ hybridization (FISH)

FISH assays were performed to detect the location of circRNA-407 and miR-9a-5p in C6/36 cells. Cy3-labeled circRNA-407 probes and FAM-labeled miR-9a-5p probes were synthesized by GenePharma (Shanghai, China). Hybridization steps were performed using a Fluorescent In Situ Hybridization Kit (GenePharma, China) according to the manufacturer’s instructions. Nuclei were counterstained with 4,6-diamidino-2-phenylindole (DAPI). Images were captured by an inverted fluorescence microscope (ECLIPSE Ti-U, Nikon, Japan). The sequence of the circRNA-407 probe for FISH was Cy3-5′-CGGCAGTTTCACTACAGCTACTCGGCTGCCTGTTTCCAAA-3′. The sequence of the miR-370-5P probe for FISH was FAM-5′-TCATACAGCTAGATAACCAAAGA-3′.

### 4.17 Statistical analysis

All experiments were carried out in triplicate. Data are presented as the mean ± standard error of the mean (SEM). Data were analyzed by two-tailed Student’s *t*-test or one-way analysis of variance (ANOVA) using IBM SPSS Statistics 23.0 software. *p* < 0.05 is considered statistically significant: *p < 0.05, **p < 0.01; ***p < 0.001, ****p < 0.0001. N.S., no significance. Different letters denote statistical significance at *p* < 0.05.

## Supporting information

S1 Data Excel spreadsheet containing, in separate sheets, the numerical values that were used to generate graphs, histograms etc. for Figure panels 2B, 2C, 2D, 3D, 4D, 4E, 4F,4G, 5A, 5B, 5C, 6A-6B, 6C-6D, 6E-6I, 6J, 7A, 7D-7E, 7G, 7H, 8B, 8F-8G, 9B-9C, 9D, 9F-9G, 10C, 10E, 10G, 10H, 11B, 11D, 11E, 11G and 11H. (XLSX)

S1 Table Primers and oligo used in this study. (XLSX)

S2 Table CircRNA sequencing data. (XLSX)

S1 Fig Sanger sequencing of BSJ of selected sex-biased circRNAs amplified by divergent primers. **(A)** and **(B)** Sanger sequencing confirmed the existence sex-biased circRNAs in females (A) and males (B). Red inverted triangle indicates the BSJ.
(TIF)

S2 Fig Overexpression of circDSX in C6/36 cells. **(A)** Schematic of circDSX overexpression plasmid. Expression of circDSX was driven by AePUb promoter and circularization of Aal *doublesex* exon2 was facilitated by *Drosophila DNAREP1-DM* flanking intron. **(B)** Overexpression of circDSX in C6/36 was confirmed by qRT-PCR compared with C6/36 transfected with AePUb-RFP plasmid using BSJ-overlapping primers. Relative expression of circRNA-407 in AePUb-RFP transfected cells was set as 1. qRT-PCR reactions were performed triplicates with three biological replicates and data was shown as means ±SEM. Student’s t-test was used to compare the means between two groups. ****p < 0.0001. Agarose gel electrophoresis showing overexpression of circDSX in C6/36 compared with cells transfected with AePUb-RFP. Expected bands were amplified by BSJ-spanning primers. *AalrpS7* was used as an endogenous control.
(TIF)

S3 Fig Evaluation of coding capacity of circRNA-407. **(A) Left panel:** Full mature sequence of circRNA-407. The sequences of the putative ORF were shown in red and the putative untranslated region was shown in green. **Right panel:** Schematic of putative ORF in circRNA-407. The location of start codon and end codon were shown. **(B)** Schematic of plasmid set for detecting circRNA-407 encoded protein. **Upper lane:** AePUb-circRNA-407-V5, sequence of V5 tag (grey) was divided to both sides of AePUb-circRNA-407. **Lower panel:** Plasmid for positive control of evaluation of coding ability of circRNA-407. The putative ORF sequence of circRNA-407 was cloned downstream of AePUb promoter with a V5 tag infused to the C-terminus. **(C)** Sanger sequencing confirmed the accurate circularization of AePUb-circRNA-407-V5. **(D) Upper lane:** Western blotting determined the coding capacity of circRNA-407 using antiV5 antibody. **Lower lane:** β-actin protein determined by western blotting as an endogenous control. C6/36 transfected with AePUb-RFP was used as a negative control (NC).
(TIF)

S4 Fig Dynamic expression levels of miRNAs in fat body of female mosquitoes after blood meal and dead-RfxCas13d-sgRNA mediated RNA immunoprecipitation. **(A)** to **(E)** Expression levels of putative circRNA-407 interacting miRNAs in female fat body during oogenesis determined by qRT-PCR. All qRT-PCR reactions were performed triplicates with three biological replicates and data was shown as means ±SEM. **(F)** Western blotting showing the translation of putative deadRfxCas13d protein using antiV5 antibody. *β-actin* protein determined by western blotting as an endogenous control. **(G)** Western blotting showing the efficiency of Dead-RfxCas13d-sgRNA mediated RNA immunoprecipitation using the AntiV5 antibody.
(TIF)

S5 Fig Sanger sequencing of pmirGLO dual-luciferase expression vector and confirmation of circRNAs BSJ amplified by divergent primers. **(A)** and **(B)** Sanger sequencing of the putative miRNA-9a-5p binding site and mutated version in circRNA-407 (A) and *Foxl* (B) of pmirGLO dual-luciferase expression vector respectively. **(C)** and **(D)** Sanger sequencing confirmed the existence of circRNA-48 (C) and circRNA-306 (D) in RfxCas13d-sgRNA-mediated circRNA knockdown. Red inverted triangle indicates the BSJ. **(E)** to **(H)** Sanger sequencing confirmed the existence of *D*. *melanogaster* homologous circRNAs in *Ae*. *albopictus*. Red inverted triangle indicates the BSJ.
(TIF)

S6 Fig Exogenous expression of an artificial circRNA in *Ae*. *aegypti*. **(A)** Linear sequence of the artificial circRNA. The 5’ end and 3’ end were colored with red and blue respectively. **(B)** RT-PCR confirmed the expression of artificial circRNA in female *Ae*. *aegypti* using divergent primers compared with mosquitoes injected with AePUb-RFP plasmid. *Ae*. *aegypti* ribosomal protein S7 gene (*AaerpS7*) was used as endogenous control. **(C)** Sanger sequencing of the BSJ confirmed the accurate circularization of the artificial circRNA. Red inverted triangle indicates the BSJ. Colored background indicates the sequence corresponding to S6A Fig.
(TIF)
