# Supplementary figures and images for: Aal-circRNA-407 regulates ovarian development of Aedes albopictus, a major arbovirus vector, via the miR-9a-5p/Foxl axis
Source: PLoS Pathog. 2023 May 5;19(5):e1011374. doi: 10.1371/journal.ppat.1011374 (PMC10191370; doi:10.1371/journal.ppat.1011374)

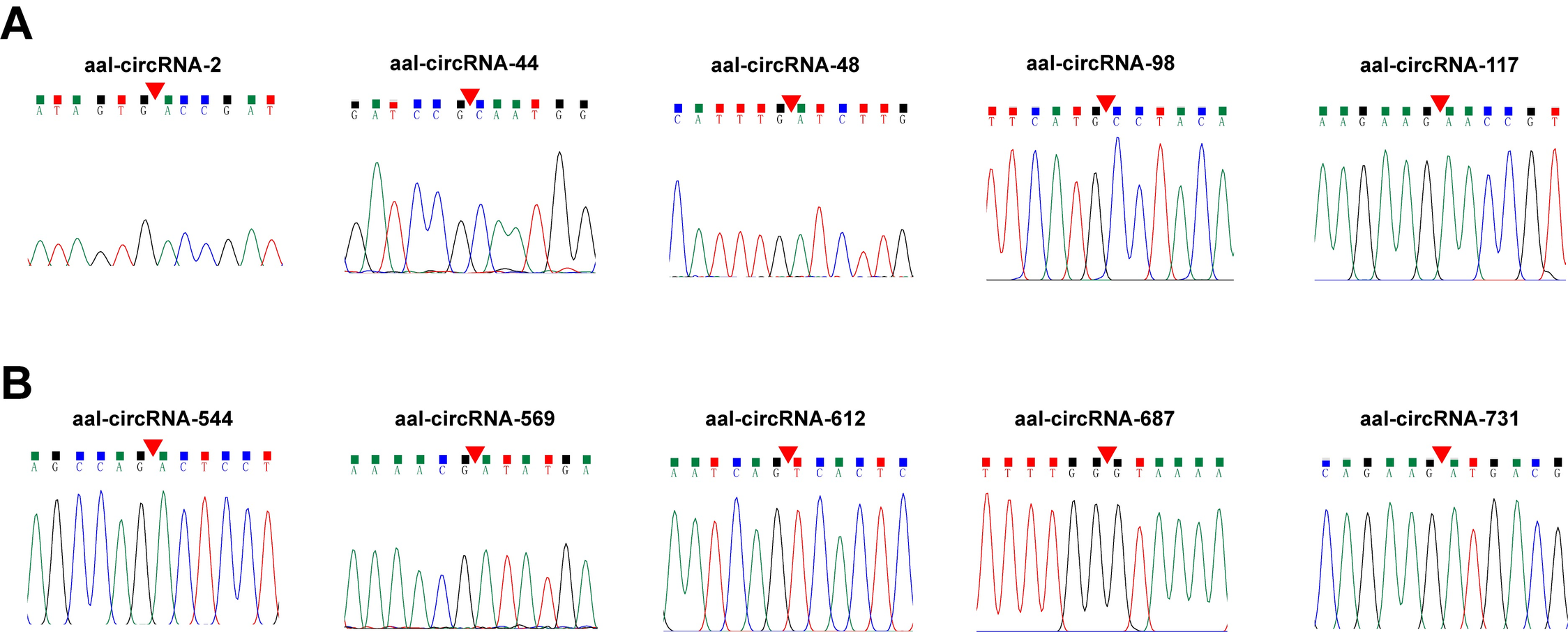

Supplement: S1 Fig — (A) and (B) Sanger sequencing confirmed the existence sex-biased circRNAs in females (A) and males (B). Red inverted triangle indicates the BSJ. (TIF) [file ppat.1011374.s004.tif]

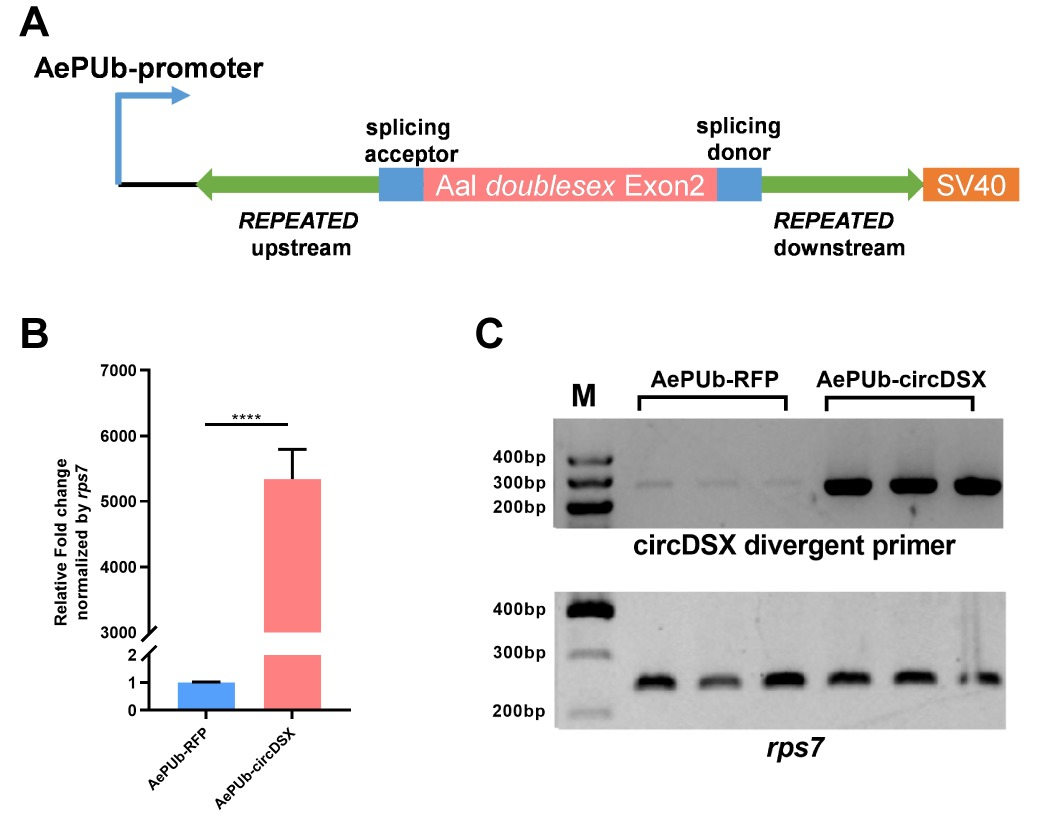

Supplement: S2 Fig — (A) Schematic of circDSX overexpression plasmid. Expression of circDSX was driven by AePUb promoter and circularization of Aal doublesex exon2 was facilitated by Drosophila DNAREP1-DM flanking intron. (B) Overexpression of circDSX in C6/36 was confirmed by qRT-PCR compared with C6/36 transfected with AePUb-RFP plasmid using BSJ-overlapping primers. Relative expression of circRNA-407 in AePUb-RFP transfected cells was set as 1. qRT-PCR reactions were performed triplicates with three biological replicates and data was shown as means ±SEM. Student’s t-test was used to compare the means between two groups. ****p < 0.0001. Agarose gel electrophoresis showing overexpression of circDSX in C6/36 compared with cells transfected with AePUb-RFP. Expected bands were amplified by BSJ-spanning primers. AalrpS7 was used as an endogenous control. (TIF) [file ppat.1011374.s005.tif]

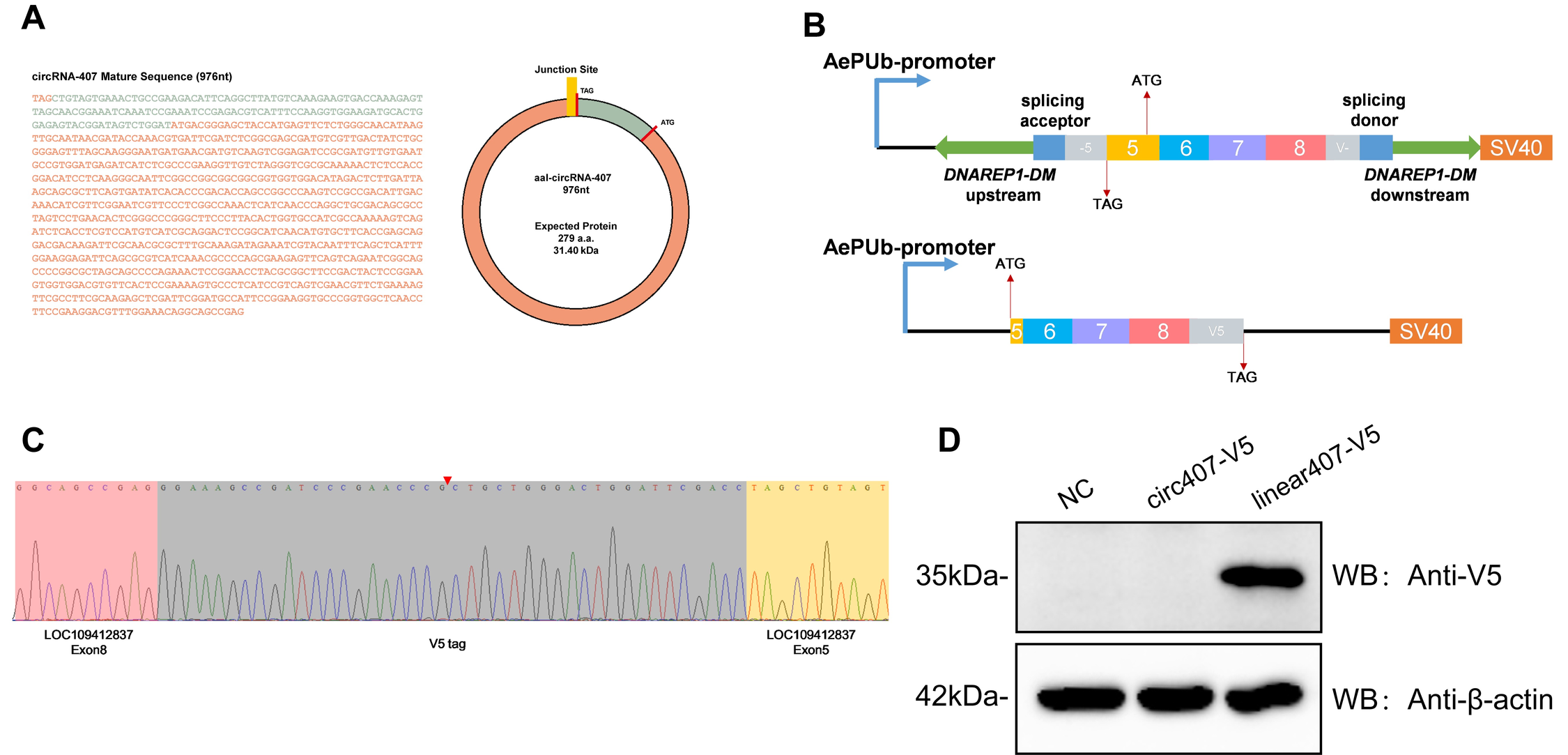

Supplement: S3 Fig — (A) Left panel: Full mature sequence of circRNA-407. The sequences of the putative ORF were shown in red and the putative untranslated region was shown in green. Right panel: Schematic of putative ORF in circRNA-407. The location of start codon and end codon were shown. (B) Schematic of plasmid set for detecting circRNA-407 encoded protein. Upper lane: AePUb-circRNA-407-V5, sequence of V5 tag (grey) was divided to both sides of AePUb-circRNA-407. Lower panel: Plasmid for positive control of evaluation of coding ability of circRNA-407. The putative ORF sequence of circRNA-407 was cloned downstream of AePUb promoter with a V5 tag infused to the C-terminus. (C) Sanger sequencing confirmed the accurate circularization of AePUb-circRNA-407-V5. (D) Upper lane: Western blotting determined the coding capacity of circRNA-407 using antiV5 antibody. Lower lane: β-actin protein determined by western blotting as an endogenous control. C6/36 transfected with AePUb-RFP was used as a negative control (NC). (TIF) [file ppat.1011374.s006.tif]

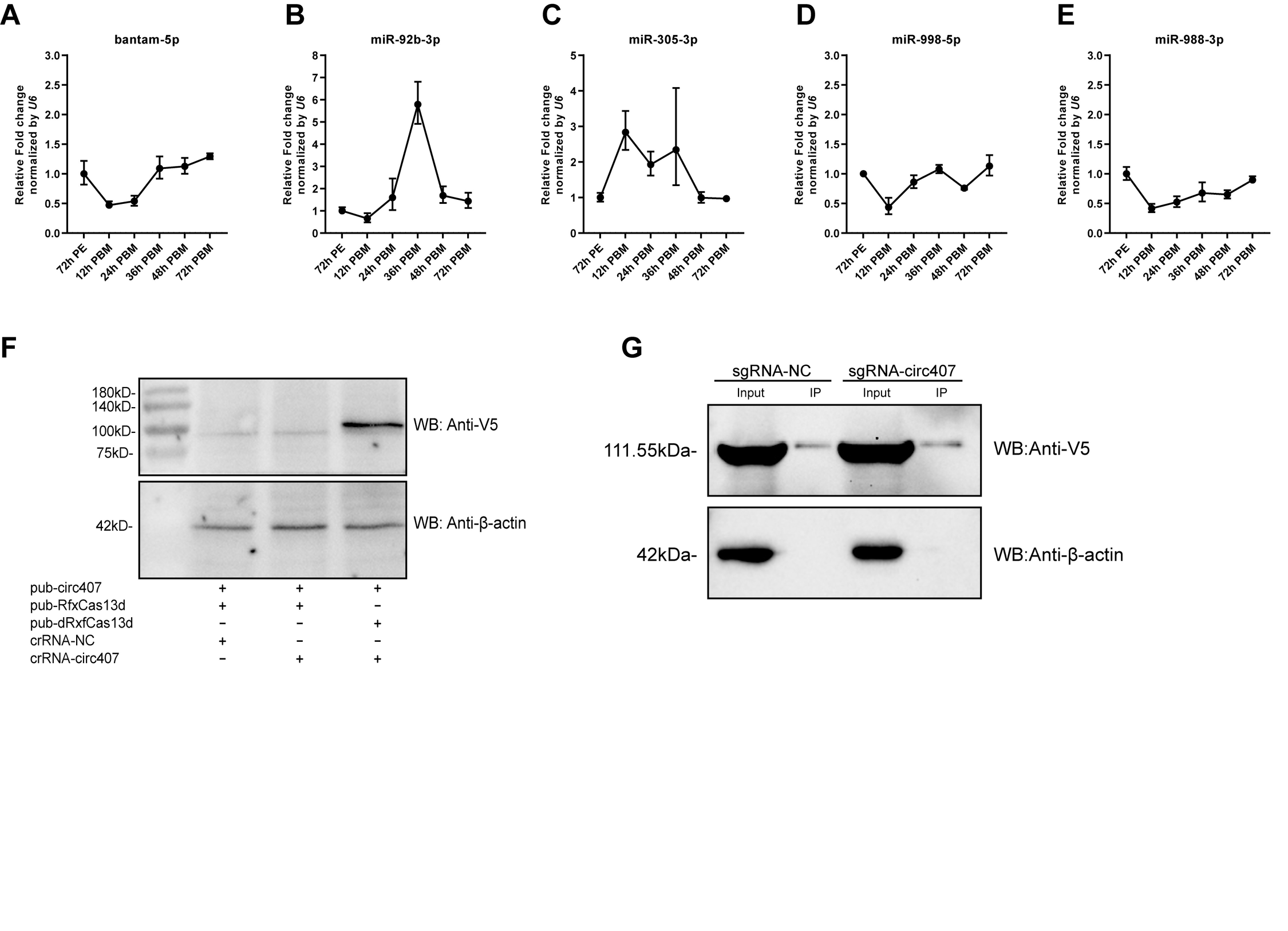

Supplement: S4 Fig — (A) to (E) Expression levels of putative circRNA-407 interacting miRNAs in female fat body during oogenesis determined by qRT-PCR. All qRT-PCR reactions were performed triplicates with three biological replicates and data was shown as means ±SEM. (F) Western blotting showing the translation of putative deadRfxCas13d protein using antiV5 antibody. β-actin protein determined by western blotting as an endogenous control. (G) Western blotting showing the efficiency of Dead-RfxCas13d-sgRNA mediated RNA immunoprecipitation using the AntiV5 antibody. (TIF) [file ppat.1011374.s007.tif]

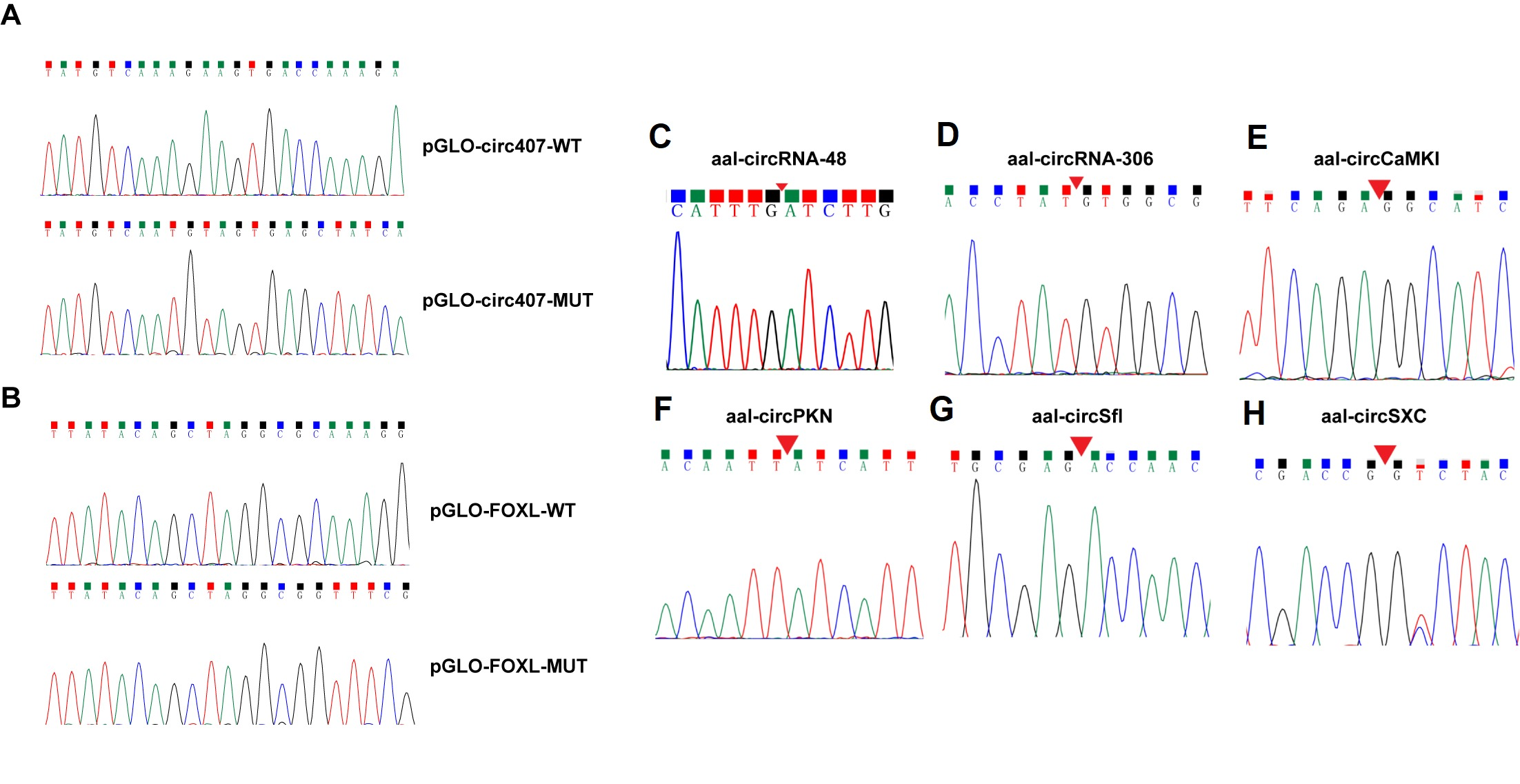

Supplement: S5 Fig — (A) and (B) Sanger sequencing of the putative miRNA-9a-5p binding site and mutated version in circRNA-407 (A) and Foxl (B) of pmirGLO dual-luciferase expression vector respectively. (C) and (D) Sanger sequencing confirmed the existence of circRNA-48 (C) and circRNA-306 (D) in RfxCas13d-sgRNA-mediated circRNA knockdown. Red inverted triangle indicates the BSJ. (E) to (H) Sanger sequencing confirmed the existence of D. melanogaster homologous circRNAs in Ae. albopictus. Red inverted triangle indicates the BSJ. (TIF) [file ppat.1011374.s008.tif]

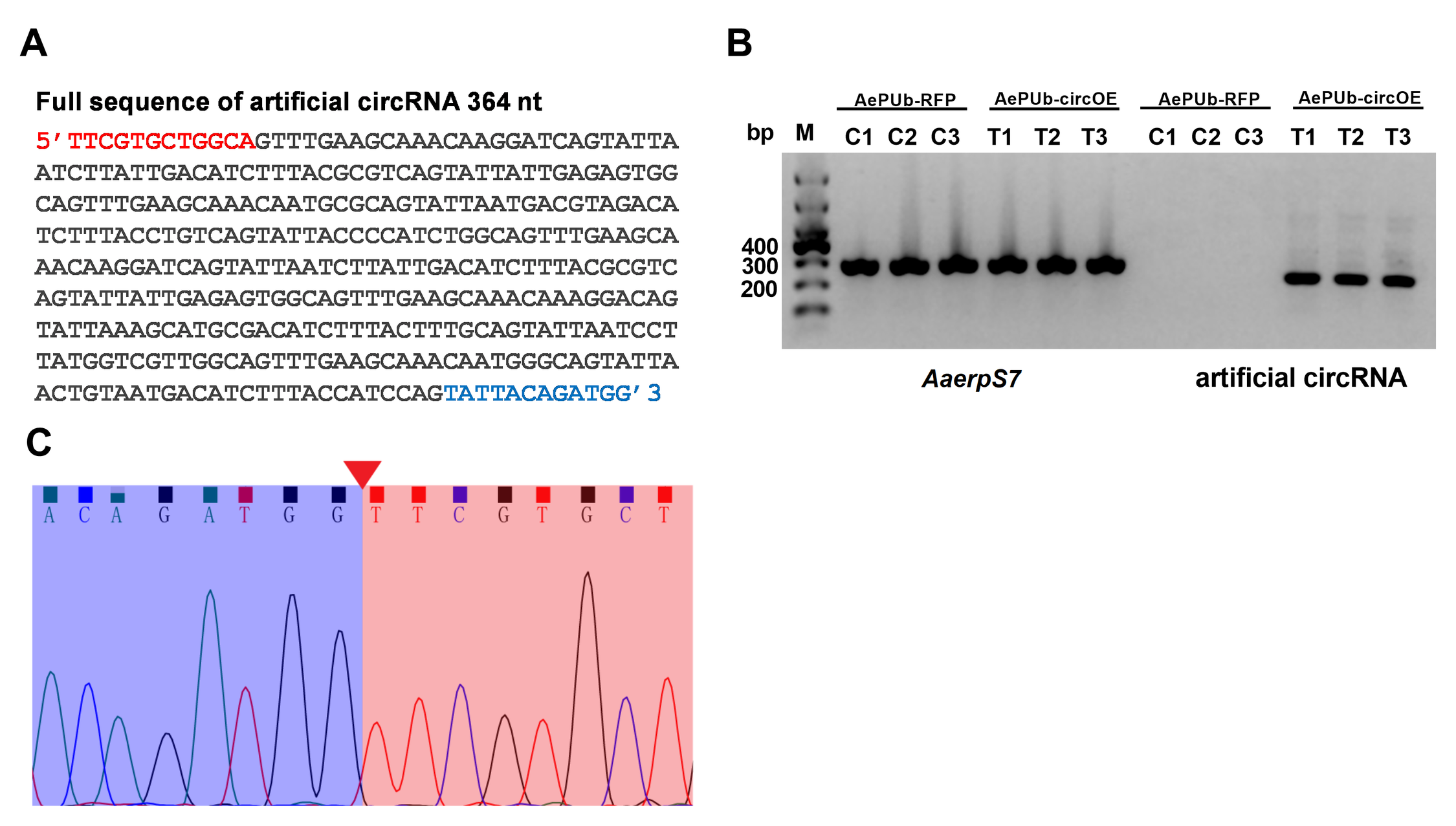

Supplement: S6 Fig — (A) Linear sequence of the artificial circRNA. The 5’ end and 3’ end were colored with red and blue respectively. (B) RT-PCR confirmed the expression of artificial circRNA in female Ae. aegypti using divergent primers compared with mosquitoes injected with AePUb-RFP plasmid. Ae. aegypti ribosomal protein S7 gene (AaerpS7) was used as endogenous control. (C) Sanger sequencing of the BSJ confirmed the accurate circularization of the artificial circRNA. Red inverted triangle indicates the BSJ. Colored background indicates the sequence corresponding to S6A Fig. (TIF) [file ppat.1011374.s009.tif]
